# Real-Time Auto-Monitoring of Livestock: Quantitative Framework and Challenges

**DOI:** 10.3390/s25185871

**Published:** 2025-09-19

**Authors:** Sarah Brocklehurst, Zhou Fang, Adam Butler

**Affiliations:** Biomathematics and Statistics Scotland (BioSS), Edinburgh EH9 3FD, UK; zhou.fang@bioss.ac.uk (Z.F.); adam.butler@bioss.ac.uk (A.B.)

**Keywords:** sensors, prediction, decision-making, prediction validation, livestock, time series, statistical modelling, latent variable modelling, machine learning, neural networks

## Abstract

The use of automated sensors has grown rapidly in recent years, with sensor data now routinely used for monitoring in a wide range of situations, including human health and behaviour, the environment, wildlife, and agriculture. Livestock farming is a key area of application, and our primary focus here, but the issues discussed are widely applicable. There is the potential to massively increase the use of empirical data for decision-making in real time, and a range of quantitative methods, including machine learning and statistical methods, have been proposed for this purpose within the literature. In many areas, however, development and validation of quantitative approaches are still needed in order for these methods to effectively inform decision-making. Within the context of livestock farming, for example, it must be practically feasible to repeatedly apply the method dynamically in real time on farms in order to optimise decision-making, and we discuss the challenges in using quantitative approaches for this purpose. It is also crucial to evaluate and compare the applied performance of methods in a fair and robust way—such comparisons are currently lacking within the literature on livestock farming, and we outline approaches to addressing this key gap.

## 1. Introduction

The use of automated sensors to collect data has grown extremely rapidly in recent years. Automated sensor data are now being used routinely for monitoring a wide range of systems, including human health, welfare, and behaviour, the physical environment, wildlife, and agriculture. Automated sensors have the potential to massively increase the use of empirical data in decision-making. This is partly because they are able to collect very large quantities of data, often at much lower cost than more traditional methods of data collection, and partly because they can collect data at a sufficiently high temporal resolution to feed into decision-making in “real time”. In agriculture, numerous recent review papers discuss sensors in the areas of precision farming, smart farming, big data, and so on. Digital transformation/smart farming for sustainable development is discussed in [1], with an overview of how new precision farming technologies assist farmers in management/decision-making, from which it is clear that artificial intelligence (AI) and machine learning play a critical role. Technologies are used to measure the environment (weather, GHG emissions), crops, soil, water, and animals. In precision farming, collected data is used for control (e.g., irrigation, fertiliser application, weeds, pests and diseases, feeding) and prediction for decision support (e.g., weather forecasting, crop and animal yields, …). Other reviews [2,3] examine recent trends in precision farming for both crops and livestock and contain tables showing a range of technologies along with the main objectives. Many review papers discuss artificial intelligence applications [3], focus on AI/machine learning [4,5], or deep learning [6].

For livestock, there are numerous recent reviews in the general area of precision livestock farming (PLF) [7,8,9,10,11,12,13,14] that discuss sensor data and quite a few review papers that focus on machine learning/AI within this field [15,16,17,18,19]. Moreover, ref. [20] suggests that much more work is needed to get to the stage of real-time auto-monitoring of livestock on farms whilst ref. [21] emphasises that development needs to be guided by livestock farmers’ needs enhancing (not substituting) farmers’ capabilities. Sensors used depend largely on the environments in which livestock are kept, with a major distinction between housed animals versus those that graze. They also depend on the practicality as well as the trade-off between the cost and value of individual animals being monitored versus animals being monitored in groups.

Because of the value, as well as the high level of management and data collection for dairy cows, this area has the most extensive use of a range of sensors and research into their application with many review papers [22,23,24,25]. Moreover, ref. [25] classifies the range of sensors used as ‘At Cow’ (includes wearable sensors such as accelerometers and Global Navigation Satellite System (GNSS) collars as well as intraruminal sensors), ‘Near Cow’ (sensors at fixed locations, includes feeding, weighing, sensors at feeding locations, imaging, and all remote real-time sensors, such as Geographic Information Systems (GIS)), and ‘From Cow’ (includes milk measurements, for example). By far the most established use of auto-monitoring in dairy is to detect oestrous and/or calving [26,27,28,29,30,31,32,33,34]. The most frequently used technology for this is accelerometers or pedometers, but other methods can be used, for example, monitoring temperature or 2-D image/video analyses, and there are studies showing that these methods generally work fairly well. There are also a range of methods for detecting lameness [35,36,37,38,39,40,41,42,43,44]. Objectives for which methods are less well-established include monitoring of dairy cows’ or calves’ health and well-being [45,46], including diagnosis of specific diseases [33,40,47,48,49,50,51,52]. Machine learning is predominant in papers [18,38,40,49,50,53,54,55] on using sensor data to predict events of interest in individual dairy cows or calves.

The main distinction with precision livestock methods for beef cattle [56] used for detecting calving [57,58,59,60] or health and welfare problems [61,62,63] is the fact that they tend to be grazing, so sensors commonly used, often in combination, are accelerometers, GNSS sensors, and weather sensors. Sensors together with satellite imagery can be used to manage production and grazing more generally for ruminants on extensive systems [64,65]. Real-time monitoring of individuals is challenging for extensive systems and particularly so for rangeland systems [11,13,59,65,66], due to practical limitations such as limited battery life for sensors on animals and the difficulty of transmitting data in real time from animals in remote locations. Sensors used for sheep [65,67,68,69,70] are also usually intended for use out in the field. As sheep are less valuable than cows, less expensive options are required for them [70]. Objectives include detecting lambing [71,72,73,74], oestrus [75], lameness [43,44], and other illnesses [76,77,78], predators [79], and changes in a range of behaviour measurements [70], including behaviour relating to ewe-lamb welfare [80], and production [81].

In contrast most methods for real-time monitoring of pigs have been developed for housed pigs [82,83,84,85], but much of this is still at the development stage, with more research needed to roll this out for use on farms for real-time decision-making, particularly at the level of individual animals. The main areas of interest are to manage production and feeding [86], to predict disease [87,88,89], and to manage the welfare of piglets [90], including detection of farrowing [91] and nursing behaviour [92,93]. Moreover, ref. [88] reviews predictions of health indicators for a range of common health and welfare problems in piglets, suggesting that whilst wearable sensors could be used, locational (sensors at fixed locations) sensors are preferable and more practical. Areas of ongoing research include recognition and tracking of pigs [94,95,96,97], automatically measuring or estimating weights [96,98,99,100], estimating behaviours [96,97,98,101], including aggression [102,103,104,105] which is of concern especially after mixing. Much of this research is based on 2D and 3D image analyses [90,106,107] for which the advantage of locational sensing must be traded off against the complexity and computational load of the methods. Sound monitoring is similarly practical, as it is cheap and also locational, but in general it cannot detect individuals. Sound can be used to detect pig vocalisations [108,109], such as coughing [110] which can be indicative of respiratory disease and welfare-related behaviours such as screaming.

The main distinction with poultry is that automatic measurement on real farms is more likely to be on groups rather than on individual birds [111]. Commercial flock management is already automated in terms of controlling the environment by automatic monitoring of a range of measures in poultry houses, such as temperature and humidity. Light schedules, layer egg collection, and provision of feed and water are also automatically controlled. But in terms of real-time monitoring of actual health and welfare using sensors, methods are still at the developmental stage, with advances needed before they could be used for commercial broiler and layer flocks [112]. Methods are generally aimed at monitoring flocks or unidentified individuals within flocks. Image and video analyses include methods for estimating weights [113], recognition and tracking of individual birds [114], detecting lameness [115,116] or sick birds [117], group activity and colocation [118], and group optical flow measured in flocks as an indicator of health problems [119]. Sound analysis include methods for detecting distress calls [120] as an indicator of general productivity and welfare, abnormal sounds indicating respiratory disease [121], and for distinguishing eating versus non-eating vocalisations [122]. Other research includes the detection of feather pecking [123] and environmental sensing to indicate specific diseases [124]. RFID [125] or accelerometers [126,127] could be used with the aim of measuring behaviour on a representative subset of birds but are unlikely to be used more extensively in commercial flocks.

This review examines quantitative approaches underlying the use of sensor data for continuous monitoring of individuals, or groups, for real-time prediction of health and welfare issues. In many areas, development and validation of quantitative approaches are still needed in order to move from the developmental stage to this intended practical application [128]. Whilst there is wide-ranging literature in this area, much of it is dominated by machine learning methods; in livestock farming, there is a paucity that provides fair, robust statistical comparisons of alternative quantitative methods and evidence that the resulting decision-making performs adequately in practice on commercial farms. That is, it must be practically feasible to repeatedly apply the method dynamically in real time on farms and optimise decisions made.

We outline (Section 2) the main sensor technologies and associated data streams that are used or being researched for real-time monitoring of livestock. We frame the research problem, outlining how quantitative analyses of these data streams can inform decision-making (Section 3). We discuss quantitative approaches that could be used for real-time decision-making from automatic monitoring (Section 4), which we refer to as prediction/decision methods, as well as approaches to rigorous statistical evaluation of the resulting decision-making process (Section 5), which we refer to as prediction/decision validation. Associated research challenges are discussed in detail with reference made to livestock farming, but many issues discussed are relevant to other application areas. We illustrate points made in Section 4 and Section 5 by data simulation to aid understanding and by examining recent studies which address real-time auto-monitoring in various livestock species (Section 6). We conclude (Section 7) by summarising some fundamental generic issues that should be considered when conducting research to more effectively exploit sensor data in order to improve decision-making within the context of livestock systems.

## 2. Sensors and Data Streams for Livestock Monitoring

A wide range of sensors can be used for monitoring to aid decision-making in real time. Table 1 shows some commonly used examples of sensors for on-farm monitoring of livestock [12,13,14,23,65,70]. Sensors commonly used may depend on species, and also on whether they are housed or outside/grazing. They are generally used for managing production, nutrition, grazing, reproduction, and/or detecting health and welfare problems. The key to precision/smart farming is that data from sensors on individuals (or groups) allow the farmer to make efficient decisions in real time to take appropriate actions such as checking and isolating or treating individual animals or groups, or changing or supplementing feed for groups.

Table 1 is not intended to be an exhaustive list of sensors available, or being developed, for on-farm use; rather, it is intended to elucidate aspects pertinent to the applicability of different quantitative methods. The choice of appropriate quantitative methods will depend on the overall design of the data collection scheme and various characteristics of the resulting data streams (see Table 2) but most importantly on who/what is being measured when, and the nature of the resulting measurements.

With regards to when, some types of sensors are on the animal (e.g., accelerometers, GNSS collars or tags), generally resulting in (near) continuous time measurements, but others are at fixed locations and so often provide intermittent measurements (e.g., monitoring feeding bouts, or liveweights from walk-over weighers). Management practices may imply, or may be designed such that some of these measurements are taken at regular intervals (e.g., 2 or 3 times per day at/or to/from the milking parlour). Other measurements may be continuous time when being measured, but with lags in data availability and gaps (e.g., when accelerometers/GNSS sensor collars need to be removed from grazing sheep or cows for data download and/or battery charging), and others may be intermittent but continuous when being measured (e.g., when an individual is in view of 2D overhead cameras, or when an individual wearing a proximity logger is close to an antenna in the field). In general, data most useful for real-time monitoring is data that is available in real time at sufficiently high temporal resolution at regular time intervals (i.e., time series). Near-continuous data at high temporal resolutions (e.g., every minute), or intermittent data, may be summarised to lower temporal resolutions (e.g., daily) resulting in regular time series data that are more practical for use in real-time monitoring. However, in doing this, care must be taken not to lose aspects of the data which may be important for detecting health issues, such as changes in diurnal behaviour [128,129,130]. Spatial data could be available at a (near) continuous spatial scale (e.g., measurements from satellite imaging), at an intermittent spatial scale (e.g., GNSS on animals), or at discrete locations (e.g., in situ environmental or weather monitors). Monitoring data from sensors where each sensor measurement at each time is at a recorded spatial location (e.g., measurements from repeated satellite imaging, grazing cows wearing collars with both GNSS and accelerometers) is referred to as spatio-temporal data. Explicit approaches for real-time monitoring that utilise spatio-temporal data could be developed but here, we focus on time series data. For simplicity, spatial measurements can be summarised into time series; for example, converting animal locations at a fine temporal scale into distance travelled over predefined intervals (e.g., daily).

Who is being measured relates to whether individuals are monitored (e.g., accelerometers on dairy cows and walk-over weighers that measure one animal at a time) or whether only groups are monitored (e.g., from automatic feeders and drinkers in pig or poultry houses). Either way, all individuals in a group may be measured or just some subset (e.g., some cows or sheep grazing on the hill may have GNSS collars and some may not; only a subset of poultry flocks will likely walk-over weigh plates). Radio-Frequency Identification (RFID) tags, sometimes called Electronic Identification (EID) tags, are commonly worn by larger animals in order to record measurements from fixed or handheld sensors for identified individuals. In some cases, measurements are made on individuals, but the individuals may not be identified in the data set (e.g., walk-over weighers for sheep or pigs, used with a marking or gate system to sort them into different feed groups). Individual identification is ideal for health and welfare monitoring of individuals but clearly monitoring of groups and of unidentified individuals in groups could still be useful, since all livestock monitoring measurements are expected to be used for indicating when animals should be checked manually.

The final major consideration is the nature of the monitoring measurements available at each data point. Often the measurements are numerical and continuous (e.g., walk-over weighers, milk yield, temperature, feed or water intake measured from automatic feeders) or discrete (e.g., step counts or counts of alarm calls in successive time intervals). Occasionally the measurements are ordinal (e.g., liveweight category for sorting into feed groups, body condition scores (BCS), or locomotion scores). Another measurement type is a classifier with no ordering, and this is most often encountered when measuring behaviours according to some predefined ethogram that will be species and/or environment-dependent (e.g., classes could be lying, standing, grazing, ruminating, walking, running, sleeping, perching, feeding, drinking, …). Sometimes binary classifiers are generated (e.g., grazing versus not grazing, lying versus standing). Furthermore, the measurements from a single sensor could be single continuous measures (e.g., liveweight, feed intake), or multivariable (e.g., acceleration in 3 directions, amounts of different gases), or even more complex (e.g., sound, image). Sometimes classifications are two-way, for example, behaviour by location classes. And finally, there may be multiple data streams per individual or group arising from different sensors, often at different temporal resolutions. Note that further processing (see below) could result in data streams that are different in nature from the original raw data.

It is convenient if the monitoring data alone can be used; however, often it needs to be used in conjunction with other information on individuals or groups. For example, behaviour will likely be affected by the amount of time spent grazing versus being housed, or the weather, whilst liveweights will be affected by days in gestation, birth, or possibly changes in diet. If other information is needed, then, for real-time monitoring, the issue of whether it is practical to record the required information accurately, and in a timely manner, on real farms needs to be considered.

The data from many types of sensors will need initial checking/cleaning before it is ready for subsequent use. How this is best conducted will depend on the details of the sensor data. For example, liveweights and liveweight changes must be within sensible ranges for the species, and can only change gradually, whereas behaviour could change suddenly. There is a range of methods that could be used for data cleaning, but it is important that these methods do not eliminate genuine data that are indicative of problems we want to detect, and that this process is automatable on-farm.

Some sensor technology has underlying methods implemented in associated software packages that automatically convert the raw data into other derived measurements, or researchers may habitually do such a conversion before further analysis. For example, accelerometer data, where the raw data is usually acceleration in three dimensions at fine temporal resolution, is often converted to a general activity measure, time budgets of behaviour classes [131,132,133], or counts of behaviours over prespecified time intervals. However, it could be advantageous to use raw rather than derived data for real-time monitoring (for example, accelerometry instead of behaviour counts), since estimated quantities could be inaccurate, and, more generally, information is likely lost in estimating derived quantities, but this must be traded off against both complexity and increased computational load. Use of all raw information might also be advantageous, as it could have the capacity to detect a wider range of problems, but on the other hand, there may be disadvantages in taking this data-driven approach, due to a lack of focus on aspects of the data streams known a priori, to be indicative of specific problems. Note that, where the use of raw data is not feasible in real-time monitoring and prediction, it would likely still be advantageous to store the raw data for validation and to develop alternative quantitative approaches. However, some devices available for use on farms only give access to derived quantities, either for commercial reasons or because on some devices the raw data are used to give derived quantities but not stored to save on memory use.

Following this, we discuss generic issues relating to real-time prediction/decision and prediction/decision validation that are applicable to any data streams, whether raw or derived, though we are focusing on time series data [134] per individual or group. As mentioned above, many sensor data streams can be converted to time series data. Furthermore, though our focus is on real-time prediction, data at fine temporal resolution may be converted to coarser resolution to reduce computational load, to smooth out noise in the data, or to align with the resolution of the prediction/decision process.

## 3. Framing the Prediction/Decision and Validation Problem

**Figure 1:**
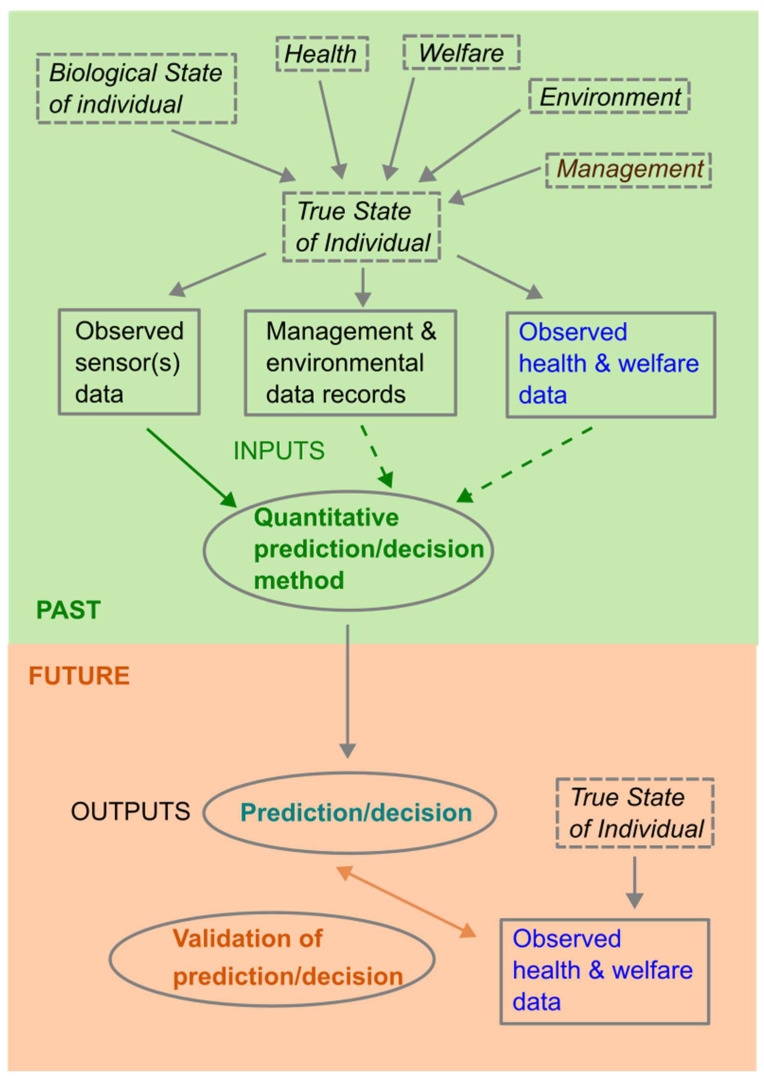
Diagram showing snap shot of prediction/decision and validation stages for an individual at one time step. Dashed arrows are optional depending on methods used as some methods are based on sensor data alone whilst others also use other observed information, for example past health and welfare data. Validation involves assessment of whether the prediction/decision made agrees with current (or near future) health and welfare data.

In framing the prediction/decision and validation problem (Figure 1), it is important to distinguish between the true (unknown) state of the monitored individual and observed data. Observed data is driven by the true state of the individual, which has multiple dependent drivers, including how it is being managed, the environment it is in, its current health and welfare, and other aspects which make up the biological state of the individual, such as breed, age, stage of gestation or lactation, etc. Observed data will be affected by noise and inaccuracies, or incompleteness due to limitations of data collection methods. In addition to this, observed sensor data may differ between individuals for other reasons; for example, normal activity in some animals may just be inherently higher, and/or more variable between times, than activity in other animals.

**Figure 2:**
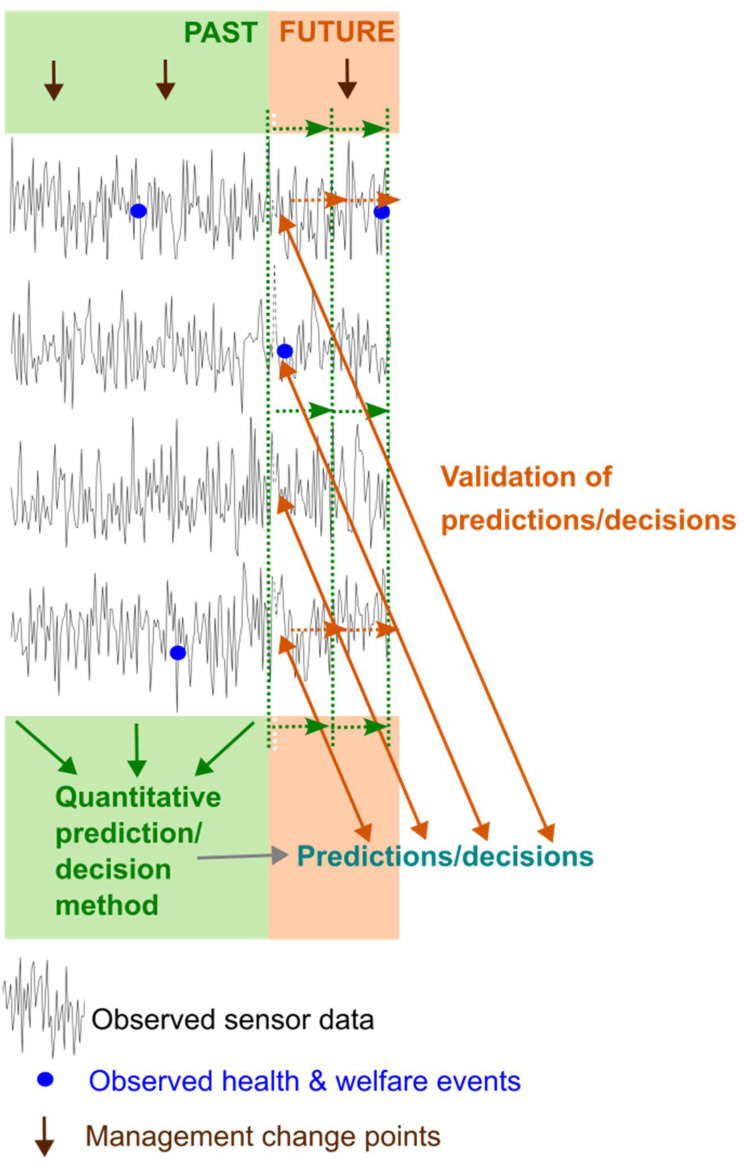
Diagram showing the prediction/decision and validation process which is repeated successively as the sensor data evolves in real time. This shows simulated sensor time series data for 4 individual animals that are being managed together. At each time step, past data are used to make a prediction/decision for each animal, which is then validated by comparing to current (or near future) health and welfare data. This process is repeated at each time step as the time series data evolves.

The objective we are focused on in the context of real-time livestock monitoring is to use past monitoring data to decide whether there is an indication that an individual (or group) has issues currently and so should be checked and managed/treated appropriately, and to be able to repeat this decision-making process dynamically in real time on farms as monitoring data evolves. All quantitative methods use some inputs (data) to obtain outputs (the prediction/decision) at each time step (Figure 1), and this process must be repeated to allow decisions to be made in real time as the data evolves (Figure 2). Some methods make predictions/decisions based on past sensor data alone, whilst others also use independently collected past observed data on health and welfare issues, and some methods may also use farm management data.

Once a prediction/decision method has been developed, it needs to be validated, which involves comparing successive real-time predictions/decisions with the health and welfare of individuals as the data evolves (Figure 2). A limitation of this is that it can only take place against observed data on health and welfare issues for an individual which are likely imperfect. Initial validation can take place on the data set on which a method has been developed but final validation must take place on other data.

## 4. Methods for Predictions/Decisions

There are numerous quantitative methods that could be applied to use available data to learn to make predictions/decisions about other data of interest, which can all be termed statistical learning [135]. The most obvious and/or commonly used methods to address the prediction/decision problem outlined in Section 3 are described below.

One important distinction between quantitative methods used to make predictions/decisions is that some of them use past sensor data alone, whilst others also use independently collected past observed data on health and welfare issues. Adopting machine learning terminology (see for example chapter 1 of [135]), we will refer to the latter as supervised methods and the former as unsupervised methods. Unsupervised methods usually involve characterising the pattern of sensor data when an individual is in a normal state, allowing unexpected data to be detected, which is taken as an indication (prediction) that an individual may have a problem and should be checked and managed/treated appropriately (decision). Supervised methods usually involve fitting a statistical model (or training a machine learning method) which uses past sensor data as inputs to predict current (or near future) observed health and welfare data as outputs. Data used for fitting could be past data from the individuals for whom we require predictions/decisions, or it could be data from other individuals. Once the model has been fitted, past sensor data from an individual may be input into the model to output a prediction of whether or not an individual currently has a health or welfare problem. For both supervised and unsupervised methods, other health and welfare data may be needed to optimise decision-making.

### 4.1. Distribution-Free Statistical Approaches

By far the simplest method for making decisions based on a numerical monitoring data stream is to use a predefined constant (upper and/or lower) threshold(s) on the current (or most recent) monitoring data to classify the unit of interest as abnormal in some way at the current time. An extension of this method would be to use different thresholds for different breeds, farming systems, and so on, or to use different thresholds depending on current environments within the same system (e.g., grazing versus not today or seasonal effects). Establishment of these thresholds will be entirely context-dependent and could be based on previous research comparing sensor data in different livestock species and systems in which a range of health and welfare issues occur.

Another generic approach to tailor this to a specific situation, or farm, would be to base thresholds on whether the current monitoring data observation lies in the (upper and/or lower) extremes(s)/outlier(s) of the empirical cumulative distribution function (eCDF) [136] of all past monitoring data collected so far on that farm. Or, to account for long-term ‘normal’ trends, or management or environmental effects that are changing locally in time (i.e., short-term effects), this could be based instead on a more recent moving window of past monitoring data that is large enough to estimate the eCDF with sufficient accuracy to judge outliers. Another obvious extension when enough data is accumulated would be to estimate eCDFs for each individual based on their monitoring data, so that what is ‘normal’ could be defined differently for different individuals. Note that this approach could allow that ‘normal’ monitoring data streams vary between individuals both with respect to means and variances. This seems a sensible approach for individuals who are usually well, but of course, it may fail to detect an individual who is constantly ill, and, conversely, may indicate an individual who is always well, as ill, just because the extremes in their eCDF do not arise from extreme monitoring data. Therefore, it is clearly oversimplistic to only consider within-individual effects. Instead, some information must be shared between individuals as well which could be done by comparing current monitoring data for an individual to its individual eCDF and the group eCDF. Note however, that any method based on eCDFs should allow that the more current observations being checked at any one time, the more likely some are to lie in the tails by chance when there is no problem. This is related to the approach based on frequencies discussed in [137].

**Figure 3:**
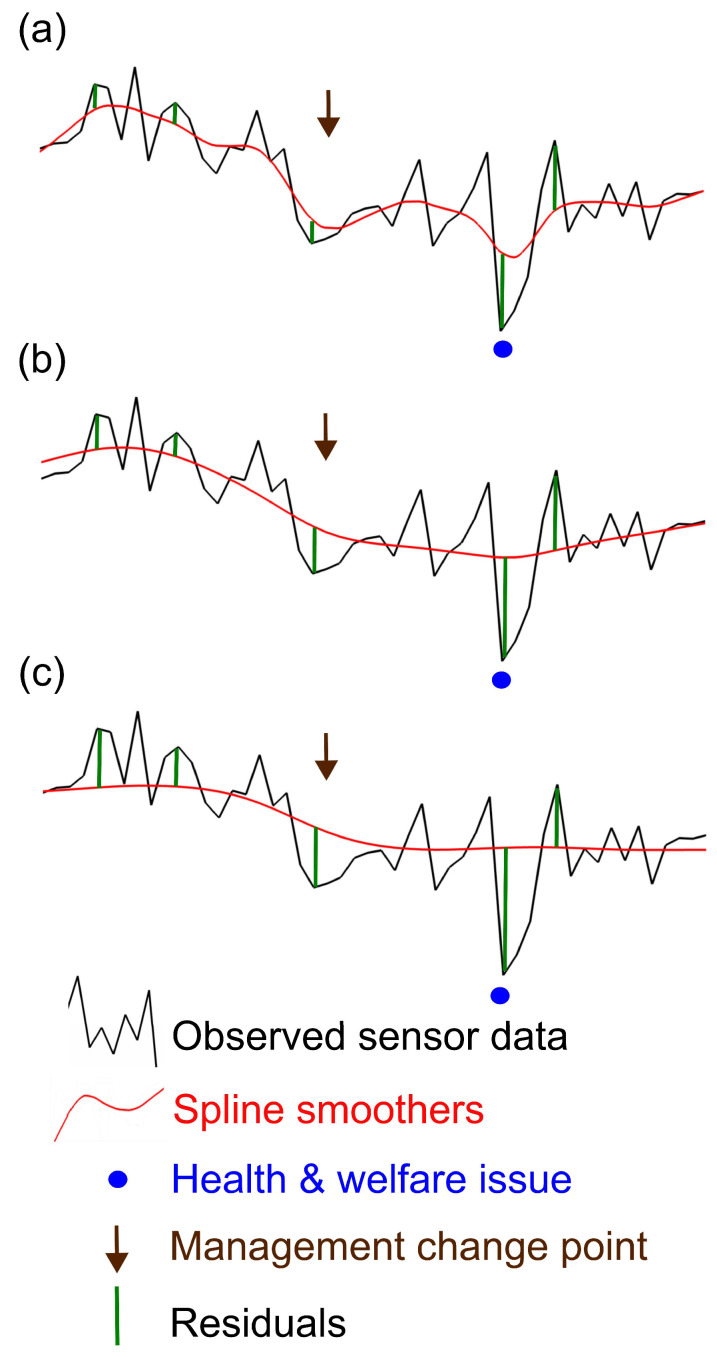
Monitoring data for one individual with (**a**) a weak spline smoother based on this monitoring data which follows short-term changes (**b**) a stronger spline smoother based on this monitoring data which follows long-term changes, and (**c**) a stronger spline smoother based on the monitoring data of all individuals managed together which thus follows long-term management group changes in time. This simulated data has one management change point, which results in a reduction in monitoring data streams from all individuals, and one later health issue for this individual which causes the sensor measurement to decrease further. The residual coincident with this health issue is relatively small for (**a**) and relatively large for (**c**), though in all three cases it is extreme compared to other residuals for this data. Here we have smoothed all the monitoring data but, for real-time prediction/decision, residuals at the current time point must be calculated from a smoother only based on past data.

Another way of dealing with long-term trends would be to smooth each monitoring data stream to establish a baseline and then to use threshold(s) to identify (upper and/or lower) extremes/outliers in the eCDF of the residuals from the smoother (i.e., the monitoring data observations—smoothed values) (Figure 3). Smoothers such as polynomial curves, simple moving averages, spline smoothers, or exponential smoothers could be used [134,138]. In deciding on appropriate smoothers and on the extent of smoothing, it is important to consider the nature of departures that would be picked up. If the degree of smoothing is too weak, short-term fluctuations indicative of a problem could be included in the baseline and so go undetected (Figure 3a). On the other hand, if smoothing is stronger (Figure 3b), longer-term fluctuations that are not indicative of a problem could be excluded from the baseline and thus may be judged to be a problem. This method may be extended by adjusting for either long- or short-term trends seen for all individuals being managed together at the same point in time, from the empirical distribution of residuals for each individual (Figure 3c). This is appealing as a generic sensor data-driven way of dealing with management effects, such as changes in feed or grazing regime, or management group (e.g., herd or flock) wide treatments, without needing to use recorded data on management, and can be useful when management effects on sensor measurements are consistent across the group. However, group-wide changes should always still be flagged, as they could indicate a herd-wide problem such as heat stress or disease spread, in which case recorded management data would still be needed to establish whether group-wide changes are due to management or indicative of some problem. Smoothing methods should generally be applied, adjusting for both management group effects and differences in individual monitoring data of individuals in their normal state.

It is possible that some basic preprocessing of monitoring data before these methods are applied may improve them. A transform such as calculating logs may improve accuracy where effects are multiplicative on the raw scale and hence linear on the log scale. Some aspects mentioned above could be dealt with by preprocessing; for example, at each time point, subtracting the mean monitoring data over the group from individual monitoring data to adjust for group-level changes, or standardising monitoring data for each individual could adjust for individual bias and noise, which may vary between individuals in their normal state.

### 4.2. Anomaly/Change-Point Detection

Detection of change points in time series data is a well-known methodological research area with a wide range of applications [139] which can be viewed as changes in the parameters underlying a statistical model of the time series. Often change-point detection focuses on the detection of one or more long-term state changes in a single univariate time series when the full series is available (offline change-point detection). However, we are interested in detecting recent changes in any of a multitude of time series based on data so far (online change-point detection). Furthermore, we are interested in detecting both abrupt short-term changes (anomalies), as well as more gradual short-term changes that could be indicative of a problem, whereas change-point detection tends to focus on non-transient changes from one underlying state to another.

The area of process or quality control [140] is relevant, in which control charts can be used to ascertain if a monitored process is out of control. This is generally implemented by generating an alarm when a measured quantity deviates from a predefined acceptable target distribution. One method commonly used is referred to as a cumulative sum (CUSUM) control chart, in which the cumulative sum is a value that can be adjusted by long-term average trends in order to ascertain whether recent values are outliers according to some predefined threshold.

Anomaly, or outlier, detection [137] applied to time series [141,142] can include methods arising from data where each data point is pre-labelled as normal or abnormal, referred to as supervised, but unsupervised anomaly detection is more commonly used. For anomaly detection, in theory, any method for forecasting time series [143] could be used, defining anomalies as time series observations that depart from their forecasts. Actual methods used for online change-point detection, or anomaly, or outlier detection, tend to fall into one of the categories below: statistical modelling, latent class or variable modelling, or machine learning.

### 4.3. Classical Statistical Modelling

Here we discuss methods with specific underlying assumptions associated with commonly used parametric statistical modelling methods.

#### 4.3.1. Modelling Usual Monitoring Data

An unsupervised approach is to form a statistical model of ‘normal’ monitoring data for all individuals (or groups), which can be applied to all (or some window of) the monitoring data so far, and then to use this, together with current/recent monitoring data, to decide whether an individual (or group) is exceptional at the current point in time, indicating there is likely to be some health or welfare problem. Formally, the statistical modelling uses past monitoring data to provide a predicted probability density function (PDF) [136] for the monitoring data for each individual at each time, with subsequent current/recent monitoring data at the upper and/or lower extremes of this distribution likely indicative of some health or welfare problem. Such methods are standard in statistics and are discussed, for example, in [142].

For numerical monitoring data, linear mixed modelling (LMM [144]) could be used with random effects included to allow for random variability between and within individuals, whilst also incorporating appropriate fixed effects characterising management, time in the year, time relative to giving birth, and so on. Extensions might be needed, such as to allow for different underlying variability in sensor data between times for different individuals or for errors that are correlated locally in time in sensor data, as is often seen in time series data.

#### 4.3.2. Modelling Based on the Outcome of Interest

The supervised approach relies on having gold-standard outcome data. The LMM described above could be used to model the monitoring data, but with the addition of an explanatory variable of a classification of a health problem, or the severity (a continuous or ordinal variable) of a health problem (or problems). After initial model estimation, this could then be used for decision-making in real time based on comparing actual monitoring data from each individual at each time with monitoring data PDFs estimated from the model, assuming no health problem.

Conversely, a statistical model could be used that directly models the outcome of interest (e.g., a classification of a health problem, or the severity of a health problem) as a function of concurrent monitoring data. For example, generalised linear mixed models (GLMM [144,145]), with logit link and binomially distributed errors, could be used to model the binary outcome health problem/not as a function of concurrent monitoring data. LMMs could be similarly used for modelling severity outcomes. As above, random effects could be used to model inherent variability between and within individuals as well as appropriate fixed effects for management changes, for example, which might cause shifts in the monitoring data and hence in the outcome versus monitoring data relationship. After initial model estimation, this could then be used for decision-making by using thresholds on the estimated probability of the binary outcome, or PDF of the severity, in the model estimated repeatedly for each individual at each time from past monitoring data together with other fixed effects.

For all these approaches, though some generic model development could be carried out for specific sensors on specific species, at some point this model would almost certainly need to be fitted and validated in the specific context in which it is to be used (i.e., for the actual animals on the farm). In particular, where there is between-individual variability in monitoring data, random effect estimates would need to be obtained for the levels of individuals in order to use this for decision-making on the farm for those individuals. Further, for methods involving modelling the outcome of interest, gold-standard data would need to be available on the farm at least initially, and possibly later on as well, to intermittently sense-check that the estimated model is still working, or to adjust it.

### 4.4. Latent Class or Variable Modelling

Latent class or variable modelling [146] can be used to model monitoring time series as a function of either unobserved states (e.g., the individual has a health problem or not) or unobserved variables (e.g., the severity of a health problem). Hidden Markov Models (HMM [147]) are a special case of latent class modelling commonly used to model hidden mutually exclusive states underlying time series data, that could be applicable here. Cluster analysis [148] is also used to deduce latent classes, though it is limited in that it does not allow other aspects of the data to be incorporated or modelled.

It is helpful to take a more generic approach of latent class or variable modelling. Multiple mutually exclusive latent states, multiple 2-level latent state classifications, or multiple latent variables could be used for multiple health problems. In a Bayesian model [149], this can be conducted by specifying a parametric model for the association of the numerical monitoring data to the unobserved state(s) or variables. Bayesian modelling [150] also provides the flexibility to model the latent class(es) or variable(s) in combination with other fixed and random effects as described above. Furthermore, relationships between the latent classes/variables and covariates could be included in the model, for example, to include the tendency of health issues to occur at particular times of year (e.g., lameness in grazing animals) or times relative to birth (e.g., health issues that tend to occur soon after calving in dairy cows).

The resulting posterior distribution of the latent class(es)/variable(s) provides an estimate of the probability that an individual has a health problem, or an estimated PDF for severity, at the current time, which could be used for decision-making.

This approach can be classed as unsupervised in the sense that no data on the health status of individuals over time is needed for its application. Theoretically it is the most obvious direct approach to this problem. However, whilst it is relatively easy to specify appropriate models in a Bayesian framework, repeated fitting of the model as the monitoring data evolves for multiple individuals is likely to be computationally intensive, though algorithms have been developed [151] for sequential data that are specifically designed to address this kind of problem.

### 4.5. Machine Learning Methods

Machine learning is widely used in agriculture [4,15,16,152] including in livestock production and welfare. It is the most commonly used prediction method in recent papers for predicting health problems in livestock from auto-monitoring data [37,40,45]. Moreover, [135,153,154] give introductions to machine learning methods and overviews of different techniques. Basically, machine learning is based on data that contains data points with multiple features (measurements that can be numerical or categorical). The machine learning task will consist of using one or more of these features (referred to as input features) to predict one or more other features (referred to as output or target features). Supervised machine learning is when the method is trained on a set of data points with known output features, usually referred to as labelled data points. Unsupervised machine learning methods are trained on data sets containing input features only and must predict output features, most commonly classifications, by patterns in the input features. Semi-supervised machine learning is when the two approaches are combined by application to data points, some of which are labelled and some of which are not. When the output feature is categorical (e.g., an individual has a health or welfare problem or not), this tends to be referred to as a classification problem, or clustering in the case of unsupervised learning, whereas if the feature is numerical/continuous (e.g., severity of a health or welfare problem), this tends to be referred to as regression. Regression problems can be reformulated as classifications by binning values, though this is an ordinal, not categorical, classification. Note also that machine learning can be used to predict non-mutually exclusive classes.

In general, training in supervised learning will be based on minimising some cost (or loss) function which measures the departure of the predicted outputs from the actual outputs. For example, for binary target classifications, binary cross-entropy, which is *log*(*p*) where *p* is the probability of the true outcome, summed over the data points, can be used, whilst for regression, the mean square error (MSE) can be used. Unsupervised learning will also be based on minimising some cost (or loss).

Once a method has been trained, the idea is that it can be used in other contexts for predictions of outputs, based on input features for new data points. Whether this results in accurate predictions or not depends on both whether the input features capture everything needed to predict outputs reliably and also on whether the machine learning ‘model’ holds. It may be that training is needed in new contexts in which it is to be used. Furthermore, if one of the input features needed to get accurate predictions is the identification of the individual, then the machine learning method will need to be retrained in any context with new individuals.

#### 4.5.1. Basic Machine Learning Methods

Interestingly, well-established basic classical statistical methods (such as regression, logistic regression, cluster analyses, PCA, and so on), along with parameters with the usual statistical interpretation (such as regression coefficients), are mentioned as machine learning methods in the above introductory papers. This is because, as mentioned above, all these methods are essentially doing the same thing: statistical learning [135]. That said, when used in machine learning, the methods by which the best-fitting model is found tend to differ from those in classical statistics, where methods with provable theoretical properties (e.g., maximum likelihood) are often used. Regression methods for linear relationships include ridge, LASSO, and elastic net regression. Support vector machines (SVMs) can deal with non-linear relationships by transforming the space of input features to a space with additional dimensions, in which simple linear classifiers can be used.

Decision trees and random forests are often used for supervised machine learning problems and can handle non-linear relationships. Decision trees basically involve successively partitioning the data into mutually exclusive sets based on criteria for the input features (which can be numeric or categorical), resulting in a tree that best (according to some predefined measure) separates data points in the target space, which can either be a classification (classification tree) or numerical (regression tree). It is important with decision trees not to overfit the data to the targets, as this will give inaccurate predictions on new data sets. Even if not overfitting, decision trees can appear to be good for the data set on which they are trained, as they were optimised for that, but they are often inaccurate when used to predict other data sets. To get around this, random forests are created by repeatedly generating optimal decision trees on bootstrap samples from the training data set, with each successive selection step in creating the tree based on a randomly selected subset of the input features. This results in a wide variety of trees. To get predictions, for any set of input features, estimates may be obtained for the output feature or target from the empirical PDF across these trees, on which decisions could be based.

As random forests combine trees, more sophisticated ensemble methods for combining multiple simpler models exist. For example, gradient boosting is often used as an alternative to decision trees, and can perform well. Interpretability of such methods nevertheless tends to be challenging.

#### 4.5.2. Neural Networks

Neural networks are networks that can be trained to solve a wide range of supervised machine learning problems, and they are being used increasingly recently [152]. Basically, the input features are the starting nodes, and the final nodes are the choice of output results. These output nodes usually make up a mutually exclusive categorisation (with one class per node), along with probabilities for each node, though continuous variables can be handled similarly by binning to get classes. There are hidden nodes in layers between the inputs and outputs. Each node takes a numerical value and is linked to nodes in the preceding layer using a weighted sum, plus some bias, and then a prespecified transformation to ensure that the resulting values lie between 0 and 1. Finding an optimum solution involves setting all the weights and biases in the network appropriately to minimise some cost function over a training data set. Algorithms (e.g., back propagation) underlying neural networks do this efficiently. Recurrent neural networks (RNNs) are suggested for predicting or classifying future time series from past time series. They can cope with sequences of varying lengths and contain feedback loops so that predictions for the current time depend on the past time series. The underlying model is stable in the sense that weights and biases linking consecutive values remain constant. However, due to the vanishing gradient problem, they have a particular difficulty with capturing long-term dependencies. Long Short-Term Memory (LSTM) networks address this issue with gated memory cells, a component of the model allowing selective, potentially long-term retention of information. This makes them better suited to working with longer time series.

#### 4.5.3. Application of Machine Learning in Prediction/Decision Context

Here, the objective is to predict different outcome measures, based on monitoring data (time series) so far for an individual (Figure 2), so this is how training would have to be conducted in order to get accurate predictions in practice. Given monitoring data streams for the full training set of data over a long period for each individual, this would create a huge training data set; for each individual at each time, the data to be used as input could be the full monitoring data so far, together with other input features such as individual-level variables (e.g., breed, age, …), time-level variables (e.g., weather, management changes), and so on. Traditional machine learning methods assume that the number of input features does not change, so to use the actual monitoring data as inputs, windowing would be needed so that the same amount of past monitoring data is used at every time step for every individual. Furthermore, machine learning methods generally ignore random effects [155] whereas it is plausible, as mentioned above, that accurate predictions of health outcomes need to consider differences between individuals in their normal state. Including individuals as part of the input features (for example, in neural networks, there could be one input node binary classifier per individual) would allow this at the cost of generalisation, especially to new individuals. A compromise wherein individuals are assigned into broad categories (for example, more active, less active) or described by a few parameters, thus capturing this variation, may be more generalisable. A simpler approach could be to achieve this adjustment by appropriate data preprocessing.

Research in this area [40] often avoids these various complicating issues, for example, by reducing the data set to one (positive or negative) event per individual and summarising monitoring data, in a fixed number of multiple ways, up to that event time. More generally, a set of pertinent input features could be derived from the set of past monitoring data. For example, plausible measures could characterise recent short-term variability for individuals compared to their long-term variability, recent changes, changes per individual adjusted for diurnal effects, or for group-level management changes, and so on. This avoids complexity in the machine learning methods, but at the expense of a lack of generality, because of having to decide a priori what derivations from the initial set of input data are pertinent to the objectives. Any approach that essentially discards available information in data in this way is not ideal when extensive long-term monitoring data is available for individuals. Furthermore, these preprocessing steps applied to sensor data prior to using machine learning methods seem to be counter to a perceived appeal of machine learning methods, which is that they are black boxes, purely data-driven, flexible, and generic methods. However, it is suggested [156] that for machine learning to be successful, knowledge needs to be combined with data, and that this can aid, not impede, in the generalisation of machine learning methods. It is plausible that if the same data and knowledge are incorporated, albeit in different ways, for different quantitative methods, then differences in performance between these methods would likely be reduced.

### 4.6. Discussion of Alternative Prediction/Decision Methods

The main advantages of the distribution-free methods described above (Section 4.1) are that they are easy to compute dynamically in real time as the data evolves, and that they are generic, in that they could be applied to any set of univariate numerical data streams. It should be noted, though, that these methods are black-box, in that their output can lead to a binary decision, or in some cases, a measure of the extent to which that decision should be made, but beyond that, they do not elucidate more information about the underlying process. A common criticism of using black-box methods is that detailed inferences about the system being studied cannot be made. However, on the premise that the basic inference we want to make in livestock monitoring is just when to check animals or not, these methods, or completely black-box data-driven methods (such as some machine learning methods), are acceptable so long as they can be shown to work. That is, it must be computationally feasible to repeatedly apply the methods in real time on the farm, optimising decision-making along the way, in any contexts in which they are to be applied. However, a method that concludes, based on one farm or population of farms, that some simple threshold can be used on the monitoring data streams resulting in optimal decision-making may not translate to other populations.

**Figure 4:**
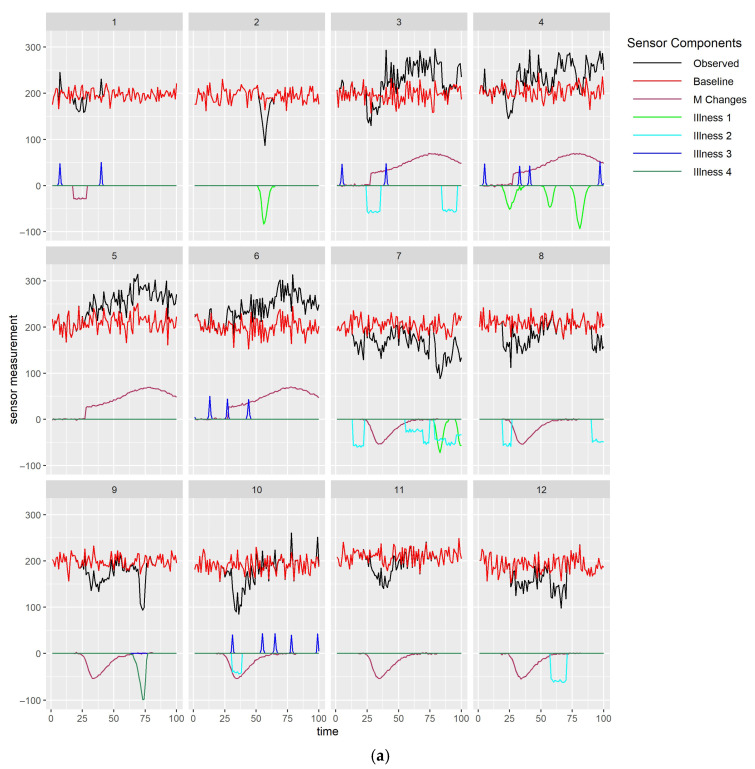

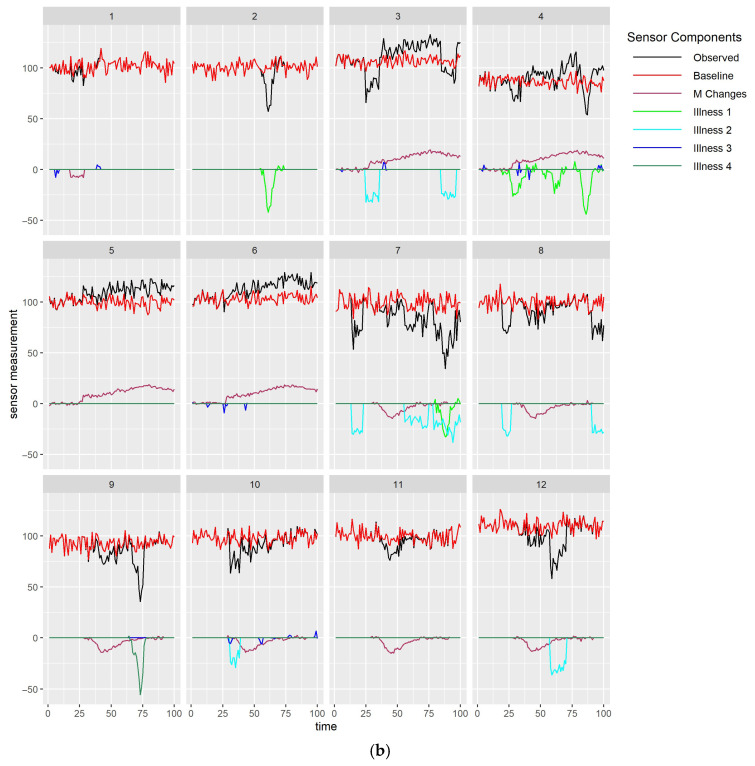
Simulated observed monitoring data (black) for two types of sensors, 1 (**a**), and 2 (**b**), together with components assumed to drive this data, for 12 animals in 3 management groups (1–2, 3–6, 7–12) for which 4 types of health issues occur. The main driver for observed sensor data is the baseline per animal (red), but observed sensor values may be altered by the occurrence of management changes or health issues. Animals in group 3–6 all show the impact of seasonal grazing; those in group 7–12 all show the impact of a diet change which reduces levels for a few weeks before recovering to previous levels whilst the management change in group 1–2 only impacts animal 1. Sensor 2 data is impacted less by the management changes than sensor 1, and for the diet change there is a delay for sensor 2 compared to sensor 1. Illnesses vary in their impact on the monitoring data, with illnesses 1 and 4 altering levels the most, followed by illnesses 2 and 3, and delayed impact on sensor 2 for illness 1. Levels are reduced for all illnesses apart from illness 3 which increases levels from sensor 1 and has negligible impact on sensor 2.

In further discussing alternative methods, it is useful to show simulated monitoring data illustrating some of the complexities to aid understanding. The assumptions underlying this simulation, and the parameters used to simulate the example data set, are described in detail in Appendix A. A benefit of using simulated data is that it allows us to generate data that would be observed as well as examine the impact of properties of underlying processes driving the observed data that would not be observed in real data. It also allows the generation of a single dataset to illustrate a range of complexities that likely occur in real data. Figure 4a,b show simulated simultaneously observed monitoring data streams from two different types of sensors (both in black), together with components assumed to drive this data, for 12 animals in three management groups for which four types of health issues occur (1&4: illnesses, 2: isolation of individual animals for health reasons, 3: heat). Observed data for each sensor depend on baselines per animal (both in red), which will depend on the current biological states of the individuals (for example stage in gestation or lactation) and which may exhibit underlying differences between animals in their average levels and noise. Baseline sensor levels (red) illustrate how some animals (e.g., 4 sensor 2) have lower levels than others (e.g., 12 sensor 2), and levels for some animals (e.g., 5, sensor 1) appear to be inherently noisier than levels for others (e.g., 9 sensor 1). Sensor levels may be altered by the occurrence of management changes or health issues. Observed data from the first sensor (Figure 4a) are behaving as we might expect for activity measured by accelerometers, with activity immediately increasing with increased grazing, and decreasing with diet changes, and health issues, apart from small increases in activity seen with heat events (labelled as illness 3). Note that activity decreases when individual animals are isolated for a health reason (labelled as illness 2), which could merely be indicative of their sudden change of environment. Observed data from the second sensor (Figure 4b) also change, though in some situations to a lesser extent, with grazing, diet changes, and health issues apart from heat, but changes in response to some of the health issues (1) tend to lag behind the changes seen for the first sensor. Slower and more gradual changes in response to management or health issues would be expected in sensor measurements such as live weight or milk yield than in sensors that measure behaviour.

Some of these complexities may make it difficult to apply methods on the farm; for example, methods need to cope with major changes that occur routinely, such as the birth of offspring, or management changes, for example, altered grazing patterns within the year, changes in feed, or routine health care. Further, if sensor data from different animals have inherently different biases or underlying variability, this must be allowed for in any method. Any of the above methods used in such a way that this is not made explicit could be applied to derived data for each individual that standardises a priori for these differences. That is, data streams could be preprocessed in such a way as to adjust for these differences. This could also make methods validated in one situation more transferable to another. In the case of supervised machine learning, this could avoid the need to retrain the method for every new situation in which it is to be used, whilst in the case of statistical and latent variable/class modelling approaches, this could result in simpler models where repeated fitting over time is computationally feasible. Statistical and latent variable/class modelling approaches, or machine learning methods, that explicitly allow for these added complexities are intuitively appealing, but could be computationally intensive or result in non-identifiability (the method is too complex to achieve robust generalisable fits/estimation given available data). However, whether this is achieved via preprocessing adjustments or via direct modelling, one important thing to consider is whether a method would require all this information to be entered in real time when this is used on real farms, as this could be impractical.

An alternative way of tackling major change events to avoid utilising this information is to simply allow the method to include them (along with health and welfare issues) in the list of issues the method is intended to detect, or include them as additional latent states or latent variables (e.g., time grazing, time relative to calving), but this would clearly increase the complexity of these approaches. Furthermore, whilst intuitively appealing, this highlights that latent class or variable approaches may need multiple classifications or variables to capture events such as these, as well as different types of health or welfare problems, making the underlying models far too complex. That said, a model that identifies different problems would be very useful for decision-making.

One disadvantage of unsupervised statistical approaches that characterise normal monitoring data in order to detect outliers from that is that once abnormal monitoring data is detected, something must be done to ensure that it does not contribute to subsequent characterisation of ‘normal’ monitoring data. Of course, it is feasible to do this so long as one is reasonably certain that the monitoring data is abnormal (and can exclude this on the farm either manually or, preferably automatically), and so long as the modelling is being conducted in such a way that there remains enough information to estimate what is currently normal for all individuals. However, it is problematic to cope with this when an unmodelled state change has occurred for an individual (such as calving). Therefore, it is more appealing to build this into the model by using latent class or variable approaches, for example, which explicitly separate normal from abnormal states continuously, and in theory could cope with additional state changes such as calving, as well as the impact of latent variables such as time of gestation or time relative to calving.

One important distinction between the various methods described above is in whether they require independent data on the outcome of interest (e.g., whether animals have a health problem, or not) for their initial estimation. Of the methods above, supervised machine learning, and some statistical modelling approaches require this, but methods that use the monitoring data alone (e.g., unsupervised machine learning, anomaly detection, latent class or variable modelling, and other statistical methods based on monitoring data only) do not. Of course, this difference is somewhat academic in the research development stage for a method, as at some point, independent data on the outcome of interest will be required to validate its performance (see Section 5) and to check that it generalises to different situations. If generalisation is not possible, methods will need to be reapplied to each new situation (for example refitting statistical models or retraining machine learning methods).

If estimated random effects (e.g., individual, farm) are needed to accurately predict outcomes for individuals from monitoring data (and cannot be adjusted for by preprocessing), then both statistical modelling and machine learning methods would need to be refitted/retrained in every context they are to be used in order to be able to make predictions for new individuals/farms. More generally, careful consideration needs to be given to whether the context in which a method is to be used is sufficiently similar to the context in which it is trained/developed.

One of the most important considerations of the more complex methods (advanced statistical modelling, latent class/variable modelling, machine learning) relates to computational difficulties for initial fitting/training and then for any refitting/retraining that might be needed on farms. The more complex methods, based on more data information, may be more flexible/generic, but are likely harder to optimise, and possibly harder to apply on the farm if the information required is not readily available. If data preprocessing and/or model simplification are required for methods in order to make them tractable, this could result in a loss of flexibility. On the other hand, more explicit modelling assumptions would be expected to improve predictive accuracy so long as they are correct. Therefore, when comparing alternative methods, it is important not to attribute differences caused by different preprocessing or underlying assumptions and trade-offs to method performance per se. Thus, for example, when input features for machine learning methods are derived via preprocessing to get around some of the complexities mentioned above, a comparison should be made with other methods applied in the same way to the same preprocessed data.

The prediction/decision problem usually results in a binary decision of whether to check an individual (or group) or not. A more generalised approach would be to view this instead as a prediction, based on past data, of a numerical quantity for each individual at each time (which is correlated with the probability of a problem or the likely severity of a disease) which could be acted upon intelligently, depending on resources, for example. Whilst there are clear benefits to this more generalised approach, including that it may be more realistic, most research in this area treats this as a classification problem. Latent variable analysis is appealing as it addresses this directly. That said, most methods described above do include additional information, in that they result in numerical estimates that carry more information, such as the probability that an individual falls into different classes, on which decisions could be based.

Another important aspect is whether the methods can handle monitoring data from multiple types of sensors or not. One simple-minded approach is to combine measures on which decisions are based together into a single index somehow, as is commonly conducted for clinical scoring or quality of life measures. This is not recommended, however, as it will likely mask important results in the individual measurements. If developed methods are based on data streams from each type of sensor, it could be relatively simple to combine the outcomes at the decision process stage. For example, if different sensors likely detect different health or welfare problems independently, a sensible approach would be to combine these by checking the animal if any of the monitoring data sources suggest there is a problem. However, if instead some aspect of the multivariable space is needed to predict a problem, methods would need to operate in this space. In theory, both statistical modelling and machine learning methods could do this directly, though undoubtedly at the expense of increased computational load.

To summarise, there are various alternative methods that can be used for making predictions/decisions (e.g., to manually check and/or treat animals) based on past monitoring data of individuals (or groups) on farms of varying degrees of complexity. The choice as to which to use in practice will need to take into account various aspects. Firstly, can they be used on a farm completely automatically with no intervention, or will additional information/intervention be needed? Secondly, is it computationally feasible to use them to make decisions dynamically in real time on the farm? And, finally, most importantly, are the predictions/decisions being made optimally? The next section discusses this latter issue, that is, how to validate the predictions/decisions from different methods.

## 5. Validation of Predictions/Decisions

Having used a method to make predictions/decisions and/or identify anomalies or change points in real time as described in the previous section, the next step in the development of these methods is to assess whether these coincide with real adverse events. We refer to this as validation. Viewed as a decision-making problem, this is an assessment of the decisions that we make dynamically as the data evolves. A decision is clearly a classification—e.g., an individual should be manually checked, as it is likely they are ill or not. However, more broadly, as previously mentioned, it can be viewed as a comparison of predictions of the extent to which there is likely to be a problem, or estimated severity of a problem, from the monitoring data, with an independent measure over time of the extent of the true health or welfare for individuals (for example, the severity of the disease as measured by some gold standard). This view of the problem is more realistic in many circumstances than the classification problem, because it is a recognition that real issues, such as disease, are usually not discrete events that happen in time, but instead likely increase over time, and then diminish again once treated. Furthermore, if the prediction is a number, rather than a classifier, the number itself could aid in decision-making in real time, for example, as mentioned above, when resources are limited, they can be focused on those individuals most likely to have the most severe problems.

It is important that animals are checked when they have a problem, preferably in the early stages of the problem, but also that there are not too many false alarms. Optimal decision-making means striking a balance in the trade-off between these two situations, and where this balance lies may be context-dependent. For example, it is more important to err on the side of caution for a serious contagious disease than it is for less serious problems such as mild lameness. Such trade-offs could be formally quantified via a cost-benefit analysis, assigning values to different outcomes such as the cost of checking animals, the benefit of identifying animals with specific illnesses, and the costs associated with missing ill animals, and then making decisions that minimise the total cost. This could also allow quantification of the benefit of sensor-based approaches compared to, or used in combination with, other approaches such as regular health check plans or preventative measures.

### 5.1. Challenges

Validation is usually not a simple problem, such as a measure of one sensor compared to the measurement of the same thing from another as a gold standard (e.g., [157]) but actually the validation of the successive predictions of either a classifier (e.g., health event or not) or an estimate of the probability or severity of an adverse event. Note that there is a parallel here with the validation of diagnostic tests for disease [158] but it is more complicated than the usual approach taken for diagnostic testing for various reasons.

Instead of one prediction per sampling unit (e.g., animal), we have a sequence of predictions so there is the additional complexity of random effects/longitudinal data, which complicates the calculation of simple validation measures akin to sensitivity and specificity.

There is often no gold-standard measurement (validation data) against which validation of predictions can occur.

Where there is useful validation data for on-farm studies, there are often lags between a problem starting and it being observed in the validation data. For example, the validation data collection may only detect the disease when it becomes severe. Indeed, if the monitoring can detect pre-clinical disease, which is often the aim of auto-monitoring, this validation data would not be expected to coincide with detection by the monitoring data. Alternatively, very accurate validation may be possible in theory, but it is not practical to collect it locally in time in a study, particularly for naturally occurring adverse events in long-term on-farm studies; for example, animals may be assessed just once a week.

Furthermore, the true issue in which there is interest in predicting will often not suddenly occur at a single time step, but more likely will gradually increase over time, and then diminish, and much, or all, of this true process may go unobserved at the time at which it is occurring.

**Figure 5:**
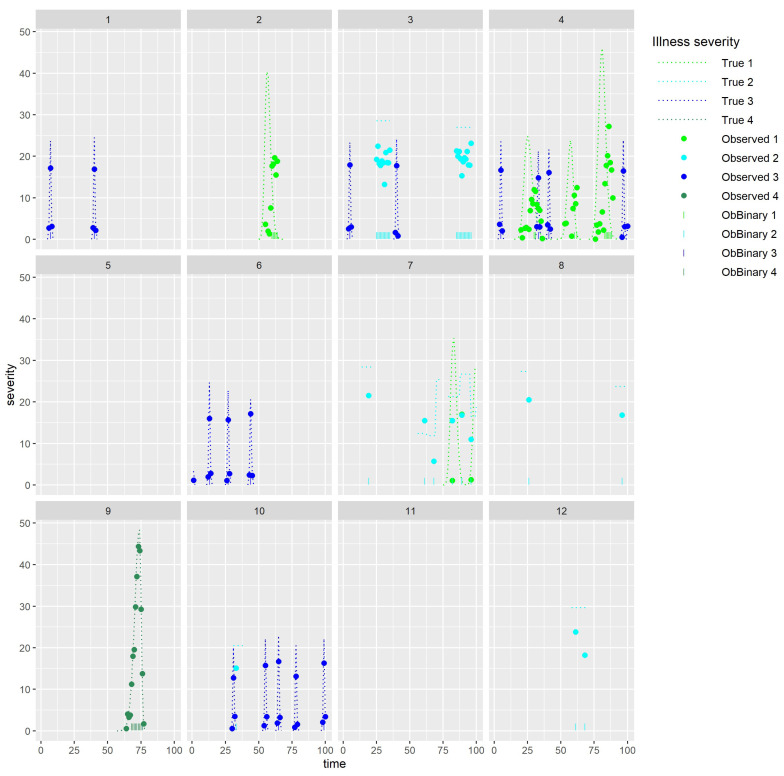
Simulated health issue severity data for 4 types of health issues for 12 animals in 3 management groups (1–2, 3–6, 7–12). The true (but unknown) health issue severity is shown as a dashed line (True). Illnesses may be gradual (e.g., illness types 1 and 4), step changes (illness type 2), or very short (illness type 3). Observed severity is shown as points (Observed). Observations available for method validation will usually be imperfect, and may lag behind the truth (e.g., illness type 1). Observed severity could be measured in some situations but more commonly the only observed data will be a binary classification (ObBinary) which may only show up when illness is severe. Furthermore, some observations may only be made at intervals (e.g., illness types 1 and 2 in group 7–12 are only observed every 7 time units) and it will be difficult to validate predictions for rare illnesses (illness type 4 only occurs once for one animal here).

Figure 5, which shows simulated illness measures for four health issues (on which the simulated sensor data previously shown in Figure 4 were based), illustrates some of the complexities mentioned above. In particular, it shows the marked distinction that likely occurs between observed illness measurement data available for validation and the true severity (one of the drivers of the sensor data).

Note that this issue of partial observation of the disease process does have parallels in the validation of diagnostic tests. A binary diagnosis may lag behind the disease process, and the true process in time for validating the test may not be observed in its entirety. Often a binary diagnosis is based on a continuous measure that has been collapsed for the purposes of decision-making using some threshold into a binary estimate for positive/negative, when in fact the initial measure (e.g., optical density) may contain more information on the probability or severity of the disease. Translation of a measure to binary to simplify the process may be sub-optimal—it could be practically useful to instead make disease control decisions optimally based on a measure that carries more information.

Another challenge is how to decide on the temporal resolution at which to validate predictions from monitoring data. If the monitoring data has been summarised prior to applying any prediction method, which may be necessary to make the prediction method computationally feasible or to simplify the prediction method, then clearly the prediction validation cannot be at a finer resolution than that. Usually, the prediction validation will operate at the same, or a cruder, resolution than the available predictions. Pragmatically, prediction validation may be limited to operating at the level at which the validation data is available.

**Figure 6:**
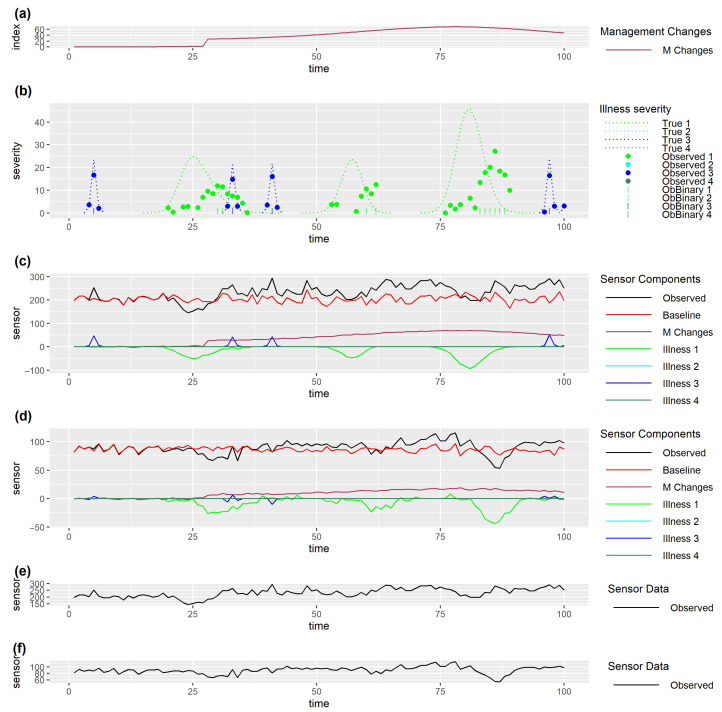
Simulated observed monitoring data (black) for two types of sensors, 1 (**e**), and 2 (**f**), together with hypothesized components (**a**–**d**) driving this data, for animal 4. Management changes (**a**) result in the overall gradual changes in sensor levels, whilst for health issues (**b**), illness type 1 results in shorter-term changes, and illness type 3 even short-term changes. Many of these health events are barely perceptible from examination of the sensor measurements alone (**e**,**f**).

Clearly, when there are multiple drivers of sensor data, it may be difficult to identify problems from the sensor data. For example, for animal 4 from our simulated data example (see Figure 6), it is difficult to visually identify many of the health issues (Figure 6b) from the observed sensor data alone (Figure 6e,f). Furthermore, the delay in the observed severity for illness type 1 (Figure 6b), together with the lag (relative to the true severity) in the change in levels for sensor 2 (Figure 6d), could lead to the incorrect conclusion that sensor 2 is better than sensor 1 at detecting illness type 1.

A lack of awareness of the prediction problem, or of the availability of accurate, timely validation data, and more generally the complexity as a result of the temporal nature of the prediction validation problem, are likely contributory factors resulting in a paucity of papers which properly tackle this to show that monitoring data and prediction methods are fit for use. Many papers [28,37,39,52,58,59,71,159,160] show associations of sensor measurements or estimates, or changes in them, with validation data which may be events or classifications in retrospect but very few address true prediction continuously in real time as described above along with rigorous statistical validation of these true predictions. Note that good association in a retrospective assessment is generally necessary but not sufficient for good prediction. There are, however, exceptions [29,40,45,51,57,72,87,91] that do address the accuracy of predictions made as the data evolves. In general, however, this is usually framed as a classification problem (often with just two classes), whereas, as mentioned above, this might be better viewed as the detection of a process characterised by one (or more) numerical values, not of state changes.

### 5.2. Data Visualisation

Due to the complexities mentioned above, data visualisation is an essential first step in the methods development process for examining the relationship between available monitoring data, or predictions from monitoring data, and the available validation data. This allows examination of how both evolve over time for individuals, especially when the two sources of data are at different temporal scales, and/or to see any time lags. Data visualisation is also essential where there are multiple types of validation data (e.g., different health problems), and multiple sources of monitoring data that might detect one or more of these problems. That said, this is a retrospective analysis to examine the feasibility of approaches. But it is more flexible than retrospective quantitative assessments such as estimates of sensitivity and specificity, or goodness of fit of the model of a numerical value, which compare monitoring data with validation data usually at the same points in time, or with predefined windows, thus oversimplifying complexities in time lags.

**Figure 7:**
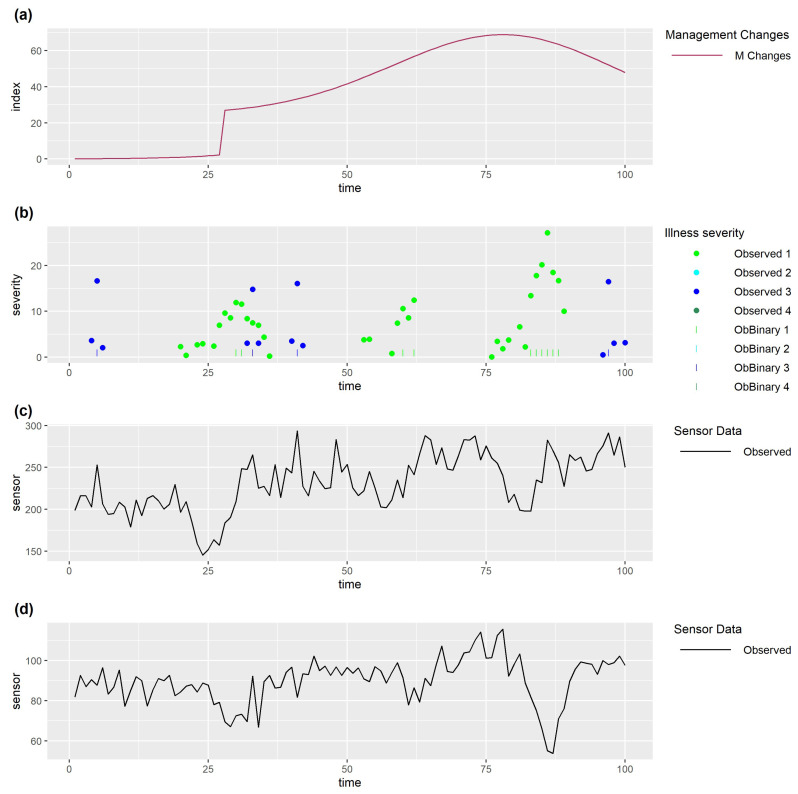
Simulated observed monitoring data (black) for two types of sensors, 1 (**c**) and 2 (**d**), for animal 4. Observed management changes (**a**) and possible observed illness data for 4 health issues (**b**) are also shown.

Data visualisation plots showing potential observed data for individual animals over time can be very informative, see, for example, Figure 7. As noted previously though, in many instances the only observed data on health issues will be binary classifications, not severity measures, and measurements of management changes may not be available.

Data visualisation plots that summarise over animals against time relative to the observed time of illness events are also useful and can be used for large data sets with many animals. However, the time of observed illness events is often not well defined, as it could be taken as, for example, per animal, the time of the first binary observation of an illness type, the time of the first positive value of an observed illness severity measure or the time of the maximum value of an observed illness severity measure

**Figure 8:**
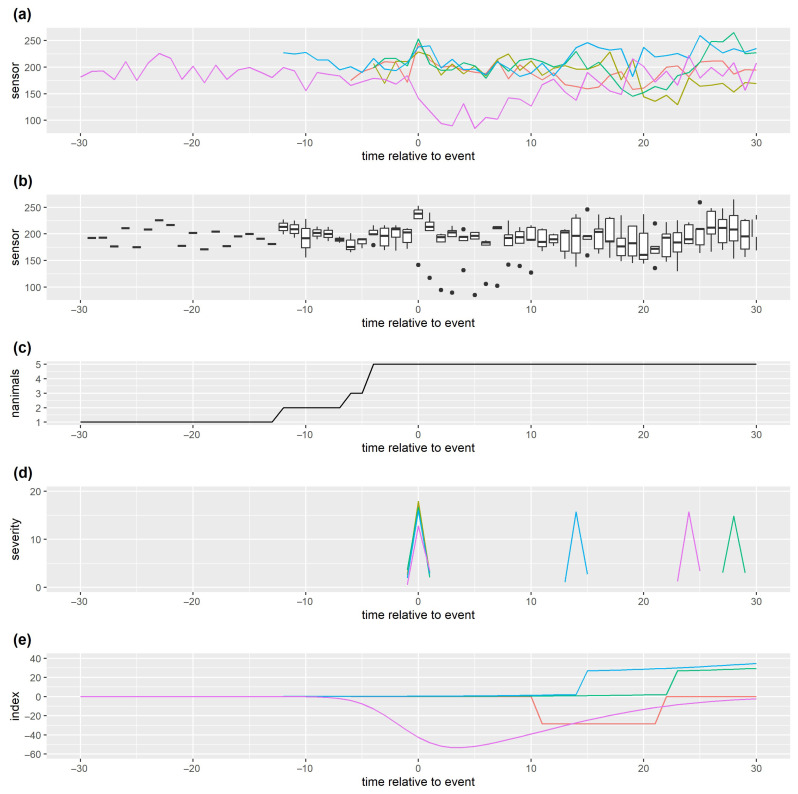
Simulated observed sensor 1 monitoring data shown in (**a**) line plots coloured by animal and (**b**) box plots over animals, plotted against time relative to the first binary observation per animal of illness type 3. Management changes (**e**) and illness type 3 severity (**d**) are also assumed to have been observed (line plots coloured by animal). The number of animals contributing to this plot (**c**) is at most 5. Both the line and box plots show that sensor 1 levels rise coincident with time 0 apart from one animal due to this being coincident with a reduction in the management index for that animal which is also driving the sensor data levels.

**Figure 9:**
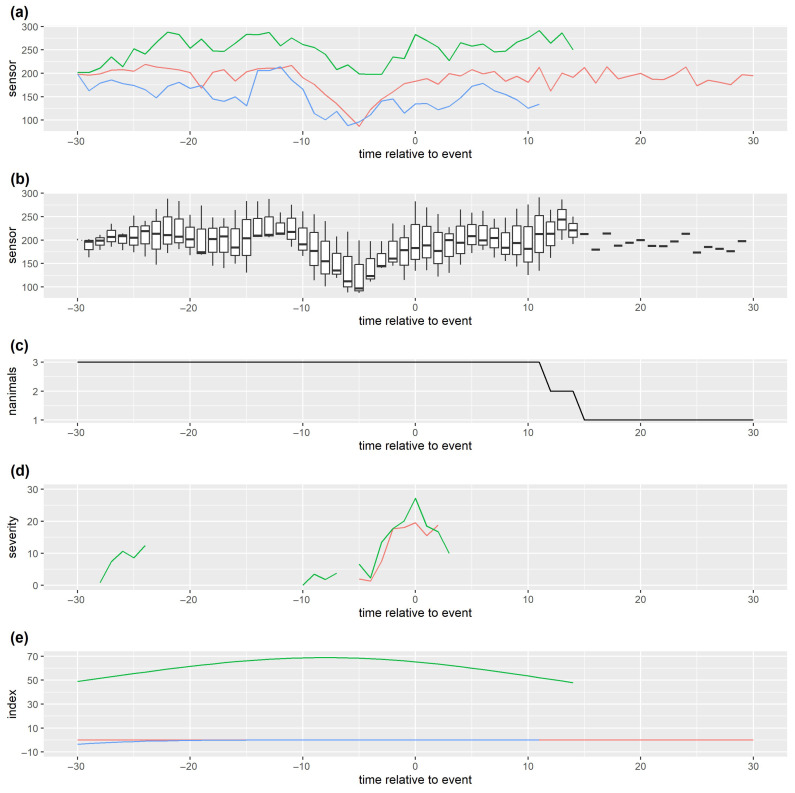
Simulated observed sensor 1 monitoring data shown in (**a**) line plots coloured by animal and (**b**) box plots over animals, plotted against time relative to the maximum observed severity per animal of illness type 1. Management changes (**e**) and illness type 1 severity (**d**) are also assumed to have been observed (line plots coloured by animal). The number of animals contributing to this plot (**c**) is at most 3. However, both the line and box plots show that sensor 1 levels show a relative (within animal) decrease prior to time 0. As mentioned previously, this is because there is a lag (of about 5 time units) in the observed severity data compared to the (unknown) true severity.

This could be plotted over all types of health issues, but since changes in sensor levels (and direction) may be dependent on illness type, patterns in plots would likely be clearer if each illness type were plotted separately. Figure 8 shows how sensor 1 levels increase at observed times of illness type 3 (which as mentioned previously would be expected for activity measurements from accelerometers in response to heat events), and Figure 9 shows how sensor 1 levels decrease ahead of observed times of illness type 1. Management changes are also shown on these graphs, though in practice on real farms these might not be recorded.

### 5.3. Quantitative Assessment

Approaches that can be taken to a quantitative assessment of predictions/decisions depend on several details associated with the context, including the nature of the measurement on which the decisions are based; whether the assessment is based on the data measurement or the decisions; the nature of the available validation data; the availability and accuracy of validation data; and the structure of the data set, in particular the hierarchy (e.g., repeated measurements within individuals within farms).

When comparing and validating predictions from multiple methods, such as parametric statistical modelling versus machine learning methods, the same goodness-of-fit statistics should be used across methods to ensure a fair comparison. Below, we discuss various quantitative approaches to validation. Observed data available for validation may itself not be an accurate reflection of the truth, but in the first instance, we put this aside and assume the truth is known.

#### 5.3.1. Classification

For testing the association of two 2-level classifications (decision to check an individual based on monitoring data versus true positive/negative state), approaches used for diagnostic tests [158] are commonly used. This consists of evaluating the sensitivity (the percentage of true positives that are checked) and specificity (the percentage of true negatives that are not checked). If the decision is based on using thresholds, a value of the threshold is commonly chosen to result in equal sensitivity and specificity, though a receiver operating characteristic (ROC) curve is usually also used, plotting the sensitivity versus 1-specificity to show the trade-off between them. Other commonly used measures of agreement, which combine information over the classes, include the phi coefficient (i.e., Matthews correlation coefficient) [161], or for more than two classes, Cohen’s Kappa [162].

All these concepts clearly need modifications in order to use them appropriately in this context. For example, they need to take into account of underlying structure. This could be conducted by averaging within individuals, and then across individuals (or farms).

Alternatives to these approaches exist, such as direct modelling by obtaining the goodness of fit from a model (GLM, say) [145,163] of the true binary outcome, as a function of the decision, or of the numerical variable on which the decision is based. These modelling approaches can easily be extended (GLMM, say) [144] to include appropriate random effects for individuals, farms, and so on. When for example, a false negative is considered more costly than a false positive, a cost-benefit-based approach could lead to more practical comparisons.

#### 5.3.2. Severity

Correlations could be used to measure the association of true severity with a numerical variable on which decisions are based. To incorporate structure, the goodness of fit could be assessed for an LMM of the true severity, as a function of this numerical variable, along with appropriate random effects for individuals, farms, and so on. Similarly, where the method only results in a classification (the decision), an LMM of the true severity, as a function of this decision (a categorical variable), along with appropriate random effects for individuals, farms, and so on, could be used. When taking a modelling approach, the usual goodness-of-fit methods, such as examination of the residuals, MSE, and so on, can be used, but it should be noted that these may be complicated by additional structure which may require careful use of weighting or averaging.

#### 5.3.3. Time Lags and Other Temporal Considerations

The more difficult issue is how to deal with time lags appropriately. One approach could just be to extend the time window on which validations are based. For example, if the method is implemented with a daily time step, validation could instead take place over some moving window of several days. Alternatively, the recorded event could be extended or moved to the day(s) before, say, which is what is effectively conducted for ‘block sensitivity’ in [40]. There is an additional issue as mentioned in [40] which is that it could be that once a health event is recorded, treatment or management decisions in response may actually alter subsequent monitoring data. In any circumstance where recording of the event itself could result in immediate treatment or management, that could impact the monitoring data, it would only be acceptable to validate predictions of events before the recording event day. Moving events to the day before (and then ignoring subsequent data) is a pragmatic way of getting around this.

These approaches, though, are fairly simplistic and involve making assumptions about likely lags. When dealing with a myriad of naturally occurring health issues on a farm, it seems unlikely that such simplistic approaches would be adequate. Furthermore, they do not really address the issue of the availability and accuracy of validation data. A more elegant and generic approach to this could be to use latent variable modelling. So here, the observed validation data, whatever and whenever it is recorded, could be assumed to be associated with a true unobserved latent variable per individual, and validation could take place instead against the estimated latent variable. However, it seems likely that different latent variables would be needed for different underlying problems. This, coupled with limitations on observed validation data (such as it only being observed at intervals and/or different types of underlying problems being rare), means that it is likely that some fairly strong assumptions that relate observed and latent variables would need to be made in order to estimate latent variables.

### 5.4. Cross-Validation

Cross-validation is usually used for machine learning methods, for which it is applied by splitting the available data into training and test data. The method is trained on the training data subset, and then validated on the test data subset [153,154]. This general principle of cross-validation is a sensible approach for all prediction methods [135]. For example, any method could be applied to the training data set, with model development, fitting, and thresholds for decision-making based on optimising goodness of fit, and then the goodness of fit evaluated on the test set using the same model and thresholds. Note that throughout, predictions made for each time step should be based only on past data. Cross-validation could be used for any of the approaches to validation mentioned above, whether based on classifications (e.g., decisions) or numerical estimates (e.g., numbers on which decisions will be based), and for any of the goodness-of-fit criteria, such as sensitivity, specificity, or correlations.

Predictive accuracy can depend on the selected training and test set, particularly when either of them is small. Therefore, both full retraining and testing should be applied repeatedly with multiple random selections of training and test sets. Both means and variation in performance should be reported to provide evidence that a method is consistently accurate. Random selections of training and test sets must be used, not selected based on optimising performance, as that leads to overfitting and underestimation of true prediction errors. A range of sizes of test sets may be used, which generally varies from 50% of the data set to a minimum of a single data point, as in leave-one-out cross-validation. Care must be taken in the proportion chosen here. Too small a test set would be computationally burdensome to calculate enough repetitions to appropriately cover the data, and may pose difficulties in handling strongly correlated data. Large test sets may leave insufficient data to train the model and provide adequate performance. If a large number of methods are optimised and compared by cross-validation, some differences between methods may arise from random chance, resulting in overfitting, so further independent validation of the best-performing methods may be needed.

When carrying out cross-validation, it is crucial that the selection of the training and test sets takes into account the structure of the data set. That is, selection should be between individuals, or groups, and, for data on multiple batches or farms, between batches or farms. This is to ensure independence of the test set from the training set, and to thus make sure that each individual validation is an unbiased estimate of performance, hence showing that something developed in one context will work in another. Carrying this principle to its natural conclusion, a method that has been developed and shown to work via cross-validation only in one context (e.g., on one farm or type of farm) may not necessarily work in another.

### 5.5. On-Farm Validation in Practice

Once a method has been validated based on available data as described above, additional research validation could take place on multiple farms, where the developed method is applied to a strict protocol, and more accurate, timely, targeted validation data could be collected. All the individuals for which an alarm is triggered by the method at a point in time, along with some other randomly selected or matched (non-alarm) individuals at intervals, could be immediately given a full health check by the farmer/researcher (blind to whether the alarm is true or false). If the method involves making a decision based on some numerical value, then sampling could be conducted to achieve coverage of that, either equally over its range or in proportion to its distribution. This could allow sensitivity and specificity to be estimated, provided enough data in each class (true positive, true negative, false positive, false negative) could be collected. However, this would likely be very labour-intensive.

A more feasible approach would be to carry out large-scale intervention studies assessing impact based only on routinely collected health, welfare, and production data, provided the interventions (how the monitoring is to be installed and utilised) could be well defined in protocols and farmers adhered to them. Sound experimental design principles to optimise studies should be used, such as applying different interventions (e.g., no sensors, sensors with a completely sensor-data-driven method, sensors plus methods that use additional farm-level data, and so on) randomly between matched farms, or different interventions being applied within farms to different groups of animals and/or in different periods. Baseline measures could be made so that the analysis showed the impact after, relative to before, the interventions. More sophisticated designs such as factorials or fractional factorials could be used to examine differences as well as synergies in the use of different sensors and/or prediction/decision methods. This high-level approach would get around many of the challenges mentioned above, but may be costly.

### 5.6. Other Considerations

The quantitative methods mentioned above could be used to compare multiple competing prediction methods for detecting the same health and welfare problems. However, when several monitoring methods are intended to be used for the detection of multiple potentially overlapping types of health and welfare problems, whether and how these should be reconciled at the validation stage needs to be considered.

## 6. Detailed Examples and Types of Studies

Here we discuss, through examples, the typical sorts of studies that could be used to develop and compare quantitative methods for real-time auto-monitoring of livestock. We mention general approaches to prediction/decision development used in cited publications, as well as the data collected and how it could be used, and associated challenges and other key aspects of prediction (Section 4) and prediction validation (Section 5). Such studies can be divided into three general categories: small-scale clinical studies on specific health issues, studies that investigate a specific on-farm or in-field site over time, and studies across multiple farms.

### 6.1. Small-Scale Clinical Studies for Specific Health Issues

These are experimental studies in which monitoring data are/could be collected on individuals along with regular detailed clinical measurements of specific health problems. These could either be naturally occurring problems or they could be studies where animals are challenged to cause specific problems.

Challenge studies [164,165,166,167,168] are often carried out to investigate disease progression, or the efficacy of vaccines or other treatments for specific illnesses in different livestock species. In [78] 20 grazing lambs were fitted with accelerometers in a crossover design in which half of them were challenged with saline and then with lipopolysaccharide a week later, whilst the other half were challenged with lipopolysaccharide and then saline. Visual behaviour scans were also conducted on a subset of the lambs. A retrospective mixed-models analysis suggested that the challenge significantly impacted behaviour as measured by accelerometers, providing evidence that accelerometers could be used to identify sickness in lambs. In [169] 27 piglets were unchallenged or challenged with *Salmonella enteritidis* or *E. coli*. Retrospective analyses showed that trends in live weights and accelerometer data were affected by the challenges compared to the unchallenged control. In [168] 20 hens wearing accelerometers in one of 5 batches were challenged using a vaccine and followed over 12 days. Retrospective analyses using LMMs suggested activity changed with challenge day, and that there was an association between daily activity and daily clinical scores of hens, though it could not be ruled out that changes in activity behaviour were due to habituation of hens to the test setting. One disadvantage of challenge studies when all animals are challenged in the same time frame is that this could be confounded with other experimental effects, due to the environment or habituation of animals to the experimental setting.

Small-scale clinical studies based on naturally occurring specific common health problems can also be used to validate sensors. In one such study [51,52], data was collected on 100 dairy calves with the expectation that 20% would develop Bovine Respiratory Disease (BRD). They were scored daily (age 8–42 days) for clinical signs whilst daily data was derived from sensors that continuously measured behaviour from accelerometers and feeding. Moving average and random forest methods were investigated based on different data inputs with validation of predictions based on a fixed window around observed (binary) health outcomes, to cope with time lags. Training and testing (cross-validation) were carried out once for the random forest method, with single estimates of a number of predictive accuracy measures given for each method. Prediction of pre-clinical BRD in dairy calves from feeding and pedometer data is also investigated in [50], where 54 out of 106 calves up to age 50 days developed BRD. Monitoring data was summarised daily to obtain means and SDs over moving windows, which were then standardised. After some preselection of input features, K-nearest neighbour clustering was trained on data for days when calves were sick (defined by clinical scoring and scans), together with data omitting 2 weeks prior to the first sick day. Some cross-validation was carried out, though it does not mention whether the selection of training and test sets is between calves, but the algorithm performs well in distinguishing sick days. The same algorithm applied to data 2 weeks prior to the first sick day shows that sensitivity is quite good up to 6 days before the first sick day. In [170] daily clinical measures of BRD were made in 231 growing bulls in a 70-day growth and efficiency trial. A process control method (CUSUM) was used to predict BRD ahead of clinical diagnosis (which occurred between days 28 and 38 in 30 calves) from a range of daily derived measures based on feed monitoring of individuals. Thresholds were needed to give optimum sensitivity and specificity but no cross-validation was carried out.

Small-scale studies for specific health issues provide an ideal opportunity to investigate the association between sensors and the progression of specific diseases as animals in these studies tend to be monitored continuously for clinical signs of the disease, thus providing ideal gold-standard data for investigating time lags and prediction validation. Often one or more clinical measurements are recorded, and they can be numerical, ordinal, and/or categorical, so consideration needs to be given to how these should be handled for model fitting, training, or validation. There are advantages in the simplicity of focusing on specific diseases with a high probability of occurrence in a well-controlled experimental set-up, though clinical studies based on naturally occurring specific health problems can be problematic as they rely on issues naturally occurring for method development and validation. Predictive analysis and validation could be carried out for small-scale studies so long as there are sufficient health events (naturally occurring or in response to challenge) and sufficient numbers of animals, and provided the independent gold-standard clinical measure had been measured at a high enough temporal resolution to give long enough time series per animal. Where sample sizes are small, data could be combined from multiple studies, provided they were sufficiently similar. However, the small amount of data available from many of these types of studies tends to limit the analysis of individual studies to a retrospective one of examining associations. However, piggybacking by adding non-invasive sensors to small-scale studies could be a very helpful, inexpensive route for early method development prior to moving on to the use of sensors in more realistic settings.

In summary, such studies can produce high-quality monitoring and validation data in a controlled environment which limits the effects that must be accounted for, but possibly at the expense of a loss of generality. They could be well-suited to simpler prediction/decision method development if there is enough data but if not, only retrospective analyses of the association between monitoring data and health may be possible.

### 6.2. On-Farm and In-Field Studies

These studies follow animals at a realistically managed location for a given period of time, providing monitoring data and health data. The form of this health data varies greatly. Some of these studies focus on specific health issues whilst others are intended to predict a range of health events.

#### 6.2.1. Dairy Farms

In [45] weekly clinical health scoring, and farm-recorded daily health events, on ~950 calves (age 7–56 days, housed 15 calves/pen) from a 2500-cow dairy herd gave weekly or daily calf status (sick or healthy) for a range of health issues. Feeding data was cleaned, preprocessed, and various associated measures were summarised at a daily level. These measurements were considered for the 6 days prior to a calf first becoming sick (with data thereafter omitted) and were supplemented with an equally sized data subset for days when calves were healthy. Training and testing (cross-validation) were carried out for 16 sets of input features by daily or weekly health data by 3 methods: generalised linear modelling and 2 machine learning methods, and single estimates of a number of predictive accuracy measures were compared. One major conclusion is that predictive accuracy is substantially better for the daily health data, as expected, since the weekly validation data is not timely.

In [40] data was collected over 40 months on 167 dairy cow lactations from a 65-cow herd. Input features were based on milking parameters, pedometer activity, feed and water intake, and body weight whilst target outputs were mastitis and lameness from treatment records shifted one day back. Extensive preprocessing was applied to monitoring data measurements, resulting in 471 potential input features, including daily, 3-day, and weekly summary statistics. Feature selection was used for mastitis and lameness separately, leading to a subset of 20 prior to the application of subsequent methods. For each health condition, the first health event per cow was considered, alongside a randomly selected day for healthy cows. Alternative sampling methods were also considered. A huge number of alternative machine learning methods were applied as well as ensemble methods that combine multiple methods. Ten-fold cross-validation was used, and predictive accuracy, including a version based on a short, fixed window size, was given along with confidence intervals over sets of results using different classifications. This served to illustrate various points, including that predictive accuracy could be very variable between methods and the detailed way in which they were applied, and more so for mastitis than lameness. Predictive accuracy varied between cross-validations within methods, and improved when using a fixed window (due to time lags). Furthermore, many of the methods, including simple ones like logistic regression, appeared to be performing equally well on average, whilst some quite advanced methods were consistently worse.

The Langhill pedigree herd, based at SRUC’s Crichton Royal Dairy Research Farm in Dumfries, Scotland, provides a key long-term data set for research into dairy cattle [171,172,173]. The herd has been selected for high and low genetic merit since 1973 and is usually maintained to have about 200 cows. The overall design of the study is a 2 × 2 factorial for genetic merit by management group, where 2 management regimes are investigated changing every 5 years or so in order to address objectives relevant to dairy farming. A huge amount of data is collected and maintained in a database by SRUC staff at the Dairy Research Innovation Centre and elsewhere. This includes much automatically collected data such as parlour data (2–3 times per day) from the automatic milking system, which includes milk yield, duration, peak flow, and position in parlour; live weight from walk-over weighers on exiting the parlour; lameness-related measures from step sensor platforms, Stepmetrix [174], on exiting the parlour; feed and water intake from HOKOs [175] from 2013; and behaviour at 15 min intervals from IceCube accelerometers [176].

Manually collected data at intervals (e.g., weekly) includes body condition and lameness scores and milk composition, and SCC. Detailed health records are made for all cows which include all health issues and treatments. Reproductive information, including heat and calving-related information, is recorded, and there is a detailed record of which group cows are in when throughout, as well as the times of start and end of grazing, and local weather data. This is an exceptionally rich long-term data set for investigating the performance of quantitative methods of monitoring and predicting health and welfare problems as well as calving and oestrus. The presence of data from multiple sensors potentially allows investigation of whether they can each be used to predict specific issues and be combined to predict a range of issues. The comprehensive manual reporting system for health and welfare events for individual cows is invaluable for prediction/decision validation. However, it also serves to illustrate many of the challenges that realistic on-farm study data presents. One is that there are a multitude of recorded issues that could affect the sensor data for an individual simultaneously, including multiple health issues or reproductive events as well as management. In fact, the detailed records on the management of all individuals serve to show the numerous adjustments (for example for cows being managed in different groups and for time spent grazing) that would need to be made on an ongoing basis to allow robust predictions/decisions from sensor data. Note that milk yield data will not exist in the dry period prior to calving, and then will diminish with days in calving, and can be noisy or missing coincident with udder health problems. Another issue of note is that, when a cow is ill, she may be put in a hospital pen, interfering with the sensor data soon after the point in time at which the prediction is required. Furthermore, in common with many on-farm studies, whilst the observed health data is extensive, it is not always timely.

It is notable that the above three studies each relate to only one farm, and in published studies [40,45] only a small window of available monitoring data was utilised per individual—just prior to selected event days. In [37] 10 dairy farms were visited every 30–42 days over ~1.5 years with lameness and body condition score (BCS) collected from all cows in milk (lactation numbers range 1–10) but only 374 had behaviour sensors (resulting in 2682 observations). Daily behaviour class time budgets, including rumination, eating, rest, and activity measures, and automatic milking system data, along with other cow and herd-level measurements and risk factors, including change in BCS, breed, days in milk, lactation number, and herd management details, …were used to predict mild lameness the same day using random forests. Behaviour measurements were adjusted by herd and by cow means (which would not be possible for true prediction). Whilst this study is not a continuous real-time prediction per individual, it did serve to show that better accuracy was achieved from using behaviour, milk, and additional farm information. A precision medicine study was conducted [160] on 22,923 observations of 5829 animals on 166 dairy herds during 2014 to 2016, focusing on multiple common health problems in dairy cows. This used a range of herd, cow (milk and BCS), and time-level measurements, giving 138 input features which were used with random forests to predict each disease type. Again, this study is not a continuous real-time prediction per individual, but served to illustrate that multiple input features were needed to predict health problems, with the sets of features varying between different types of problems. Another study [177] also focused on general health and welfare, in which 318 cows were selected to give coverage of lactation numbers and days in milk from six dairy farms in three different countries. Gradient tree-boosting was used to classify animal welfare status (good, moderate, or poor) based on behaviour from accelerometers and milk measures. Here numerous daily input features included gradients from linear regressions of monitoring data measurements over recent time windows of varying length per individual which served to measure individual, short-term changes. Most importantly, these three studies showed that lower, and more variable, predictive accuracy resulted when cross-validation was carried out between farms compared to between cows. Moreover, ref. [37] suggested this could be dealt with by applying methods to similar farms or by including additional input features that characterise differences between farms.

Dairy cattle tend to be the livestock system that is best studied in terms of broadly available monitoring data and detailed health records, especially in the housed context. This can result in very rich data sets but also in much variation in the types of data collected or utilised. That said, the complexity of the available data and the multiple real health problems that occur make both method development and validation challenging.

#### 6.2.2. Extensively Managed Cattle and Sheep

One practical issue with extensive systems is that often the animals need to be gathered to download sensor data and recharge batteries. In order to use these sensors in real time for predicting health and welfare problems, the data would need to be downloaded remotely, and the prediction algorithms applied, in real time, as the data evolves. Because of the practical difficulty of real-time monitoring of individual animals in extensive systems, and of obtaining real-time validation data, there are few studies that directly address real-time prediction for grazing beef cattle and sheep. One exception is [57], in which a range of sensors (accelerometers, GNSS, rumination algorithm, walk-over weight unit enclosing sole watering point, and weather) were investigated to detect calving in 40 cows in a 32-hectare paddock. Calving was measured every 2–3 h from 06:00 to 18:00. Limited conclusions can be drawn from this study, as a really large number of input features were used as candidates to detect just nine calvings, utilising the 7 days of data prior to the calving events. Nine cross-validations were carried out, leaving one cow out each time, and up-sampling the eight calving events during training of an SVM, and the mean predictive accuracy was given. However, the approach used here does serve to illustrate various points of interest. Weather data was included not because it was expected to affect the day of calving but because it was expected to affect monitoring data and hence the relationship between that and the day of calving. Predictions were investigated with both a daily and hourly time step, but daily performed better presumably because calving events were not recorded accurately enough to validate hourly monitoring. Multiple input features were derived for each day from each sensor type, with only those calculated for the current day used in the daily prediction of calving. However, these were used in three ways: unadjusted, adjusted to the herd mean the same day (after removing upper and lower quartiles), and adjusted to the data on the previous day from the same individual. These adjusted measures came in strongly illustrating the need to adjust for herd-level changes (such as weather and management) and that changes within individuals are an important predictor of calving. Note, however, that in order to predict health and welfare events where changes are gradual, rather than sudden, information from longer-term changes within individuals would need to be included in the input features.

A similar approach is taken in [72] in which GNSS, accelerometers, and weather data are used to predict lambing for ewes in the pasture. This is based on training and tuning an SVM based on one 14-day study using leave-one-out one ewe cross-validation (out of 8 observed lambings) and then validating the model by examining alarms generated in another 15-day study with 11 observed lambings. Extensive preprocessing of monitoring data was described in detail, as well as how pertinent hourly measures were derived for GNSS and accelerometer data, which were again unadjusted, or adjusted to flock means at the same time, or the same measures for each ewe 1 h and 24 h before. Four key input features were used in the final model: three from GNSS data (mean distance to peers adjusting to herd mean, distance to closest peer, mean distance to peers) and one (counts of posture changes) from the accelerometer data. Whilst alarms were generated around most lambings in the validation data set, there were quite a lot of false alarms prior to lambing which led the authors to point out the need for alarm thresholds to be varied depending on the context in which this monitoring is to be used in order to reach the optimum trade-off between sensitivity and specificity. To examine why there are classification errors, graphs for some ewes show how monitoring input features change with time relative to lambing. This data visualisation is highly informative, though strictly speaking, machine learning methods operate on the multiple-dimensional space of input features. Other points mentioned are that monitoring data was not actually available in real time in these studies, and the need to limit the number of input features to make the machine learning algorithm computable in real time. This method was further validated on a daily basis using another study [77] of 14 ewes, where alarms were first generated on the day of lambing for all of them apart from one ewe with vaginal prolapse, for which several alarms occurred on the days leading up to lambing.

In [74] accelerometers were used to infer grazing ewe behaviours using 121 ewes, and the huge change in (estimated) licking behaviour was assumed to indicate lambing day for individual ewes. Then a deep learning neural network was applied to the sensor data at each hour prior to lambing, with 30 input features, which were summary statistics over the last 7 days, with (estimated) time to lambing at that hour as the target output variable. This study highlights the difficulty of getting the true lambing time in order to validate predictions for extensive systems, and thus uses the same sensor data set to estimate lambing day as it does to predict time to lambing. That said, it suggests that time to lambing can be predicted to within 20 h accuracy up to 10 days before lambing, though cross-validation is not mentioned.

A long-term sheep flock study on the use of technologies for health and welfare monitoring is being carried out at a research farm at the Moredun Research Institute [178,179,180]. Technologies include EID for identification, accelerometers [181], GNSS [182,183], and BLE proximity loggers, with a subset of ewes and their lambs wearing this technology during several periods in 2021, 2022, and 2024. Objectives of this study include assessing PLF tools for early detection of parasites, mastitis, and lameness in a flock during summer grazing as well as which sensors are usable by sheep, practically speaking, and whether cheaper sensors can be used instead of more expensive ones. Health and welfare assessments of ewes and lambs were made at intermittent gathers about every two weeks including walk-over weighing, body condition, mastitis and dag scores, faecal strongyle and nematodirus egg counts, and treatments recorded. Whilst this is potentially a rich data set for assessing the prediction of a range of health and welfare problems using a range of sensors in the field, it presents various challenges. If particular sensors, or combinations of them, are able to predict specific health or welfare issues, we are reliant on enough instances of these issues naturally occurring in order to be able to assess predictions. This is compounded by the fact that only a subset of sheep and lambs were wearing technology, and they are only wearing it continuously for some periods. Furthermore, health and welfare data, though wide-ranging, is not available continuously in real time, with many assessments coinciding with periods when technology was not worn in the period at or immediately preceding the assessment. These issues preclude validation of predictions in real time, but investigation of the potential for real-time prediction from sensors of the recorded health and welfare problems is feasible using a retrospective analysis.

Extensively managed systems overall show much more patchy data coverage, dominated by issues of practicality. This is especially true for rangeland systems where substantial investment would be needed to enable real-time data collection from sensors on individual animals and where the collection of timely validation data representative of rangeland systems would be very difficult. Extensively managed systems are also affected by other factors like weather and grazing practices which complicate modelling.

#### 6.2.3. Pigs and Poultry

Methods used for monitoring pigs tend to be mainly for use in housed systems, and often locational sensors are used, especially when monitoring groups of growing piglets, where research is still at the stage of trying to be able to identify individual pigs. An exception to this is a study [91] in which accelerometers on sow ears were used for predicting farrowing for ~20 sows in crates. Of particular note here was the preprocessing which involved using measures of variance in the total acceleration for predefined window sizes, which were then smoothed and then adjusted by the same measure 24 h before, effectively accounting for any underlying sow and sow-dependent diurnal effects. Then a CUSUM method was used to generate alarms. This worked fairly well but it should be noted that this study was carried out on a few pigs, with no cross-validation. A fairly low-tech solution, which obtained feeding and drinking behaviour (but not amounts) based on RFID tag readers at feeders and drinkers, was used [87] to predict deaths/culls in three batches of 12 pens of 12 growing finisher pigs followed over some weeks. After some preprocessing, six daily measurements were derived for feeding and drinking for each pig. For each measurement, a Kalman filter was used to estimate each pig’s behaviour based on previous days and adjusted to pen behaviour the same day, and then anomalous behaviour, and hence alarms, were identified if the observed values exceeded confidence intervals. This resulted in high specificity but there were too few deaths/culls (just 4–5 per batch) to evaluate sensitivity. Other recent studies are more developmental, for example based on image or sound sensors, and whilst they suggest potential, they did not really address the problem of validating real-time predictions of health and welfare problems.

Similarly, methods for poultry tend to be for housed flocks. In commercial flocks, locational sensors are used to measure groups or unidentified individuals, intended to be a representative subset of a flock. There is a paucity of studies that validate predictions of health and welfare problems for flocks made continuously in real time from monitoring data, though some studies do show retrospectively an association of data continuously derived from sensors, with health and welfare outcomes in broilers. In [116] an experiment on 250 birds in 5 batches with a range of gait scores showed that walking speed automatically measured when they were walked down a runway decreased with increasing gait score. In [120], whole-house sound recording of 12 commercial flocks showed that spectral entropy was negatively related to distress call rates and to both current and more long-term weight gain and mortality for flocks. In [119], cameras in 31 UK and 43 Swiss flocks measured optical flow which was shown to be related to hock burn and mortality.

Compared to cattle, monitoring for pigs and poultry tends to be less developed, with poultry especially being affected by the large size of flocks relative to data collection capabilities.

#### 6.2.4. Summary

Overall, on-farm studies have the advantage of being more realistic and producing larger datasets than small-scale clinical studies. However, practicalities will limit what monitoring systems and health monitoring regimes can be employed in real production farms. For validation there are individual challenges to each experimental setting, as records of health issues may not be accurate and/or timely, and allowances have to be made for specific management practices that were not designed with easy and reliable analysis in mind. Whilst this can be partially dealt with by appropriate analysis methods that address issues such as time lags or incorporate known management changes, results may nevertheless fail to generalise to other locations.

### 6.3. High-Level Validation Studies

Large-scale intervention studies involving multiple farms (see Section 5.5) would be ideal for establishing whether or not real-time monitoring with sensors, along with methods for prediction/decisions, can really be impactful on real farms. However, we could not find any properly designed high-level on-farm intervention studies for multiple farms, nor even within farms, to examine the impact of using sensors for continuous real-time monitoring on the farms. In [184] survey data was compared between dairy farms that did not use any sensors and those that did use sensors (for monitoring at least milk yield and oestrus). Although they selected from this survey 50 farms that used sensors, and 50 with matched characteristics that did not, few differences in performance measures were found, and in any case, differences could be due to other factors, as this is an observational study. Moreover, ref. [185] describes a 3-year study in which a 900-hill sheep flock [186] is split into 2 groups managed with and without precision farming methods, and differences in economics, animal performance, and farm labour are examined. They did find some differences, but the precision farming was not utilising the sensors continuously in real time for decision-making.

Without more small-scale experimental and real farm studies validating their use, real-time monitoring systems may not be widely taken up, making large-scale intervention studies difficult to establish for emerging technologies. Furthermore, there is usually a gap between real-time monitoring systems appropriate for use in research studies and versions that would be appropriate for use by farmers on real farms, so further development of systems may be needed to roll the technology out onto real farms in order to carry out large-scale intervention studies.

## 7. Summary and Conclusions

There are a huge number of studies and review papers relating to precision livestock farming and much research development on quantitative methods to use sensor data streams on individuals or groups for prediction and decision-making in real time on livestock farms. However, detailed scrutiny is needed to ascertain exactly how the monitoring data and these methods are used and validated in published papers. It is difficult to draw on the literature on multiple studies to make fair comparisons between recommended quantitative methods, because numerous divergences likely impact their performance. This includes differences between data sets (overall design, monitoring data, and types of health issues), data preprocessing and how the processed data is used (both inputs and outputs), how quantitative prediction methods are implemented (e.g., underlying models and how they are fitted and predictions obtained, and how parameters are set or tuned for machine learning methods), how decisions are made, and how predictions/decisions are validated. Even within the same paper, quantitative methods are often applied and/or evaluated differently. Furthermore, even when there is reliable validation data available, there is a lack of evidence that quantitative prediction methods validated in one circumstance will work in another situation. In most of the studies reviewed here that investigate prediction/decision-making in real time, quantitative methods are applied on a single farm whilst the few multiple farm studies reviewed showed predictive accuracy is poor when methods developed are validated between farms. It is plausible that other studies which show poor performance when validated between different farms or farming systems remain unpublished, though the amount of work involved in such studies means it is likely that they are not commonly undertaken.

The amount and temporal resolution of monitoring data per individual that is used in each prediction will impact the results, as will the way in which this monitoring data is used. Therefore, it is important to make comparisons between different choices made at this stage and to recognise when comparisons of alternative prediction methods are confounded with this aspect. For example, statistical methods tend to be based on the full (or some recent window) past unprocessed time series data, whilst machine learning methods in papers examined here were often applied to input features that are summary statistics of past time series data per individual, possibly also adjusted by group effects. This latter approach is sensible for any method, as it can be used as a simple way to adjust individual measurements for group effects (for example, herd or flock management effects), for effects of individuals, and/or to capture recent short-term changes likely to be indicative of a problem. It also likely reduces the computational load of methods, and it could make the prediction methods more applicable in different situations. However, when any method uses these summary statistics as inputs, some quite strong assumptions are being made about what properties of the data are relevant, and the performance of the method will largely depend on the validity of these assumptions. Furthermore, the intuitive appeal to the uninitiated of machine learning is that it is flexible and can use large data sets to provide outcomes with few explicit assumptions and so ideally machine learning methods applied directly to (unprocessed) time series data should be investigated.

When researching quantitative methods to make predictions/decisions in real time, careful thought needs to be given as to whether the methods are being used in exactly the same way that they could be applied in real time for decision-making on real farms. As well as taking into account predictive accuracy, other aspects need to be considered, in particular, the advantage of only using monitoring data versus the need to use additional data on farms, groups, or individuals; the computational load of methods; and whether they need to be refitted or retrained in every new situation and/or for particular farms, groups, or individuals. Many research development studies focus on one or more specific monitoring data streams for specific types of health or welfare problems. However, when used on real farms, some monitoring data may be subject to changes due to multiple types of problems, and so the issue of sensitivity and specificity for multiple problems needs to be assessed. Some studies do develop models for general health and welfare problems, but it is highly probable that different sensors and/or predictive models would be required for different types of health and welfare problems. Furthermore, sensor data, and in particular behaviour data, will also be influenced by changes in farm management; for example, increased grazing or moving animals to different groups over time, and methods will need to account for this. Taking into account as much of an individual’s past monitoring data as possible, and also sharing past information between individuals, would be expected to improve predictive accuracy, but this will be limited in practice by computational load.

All that said, regardless of all these details, a method is viable, so long as it can be shown that it is practically feasible to repeatedly apply the quantitative prediction method dynamically in real time on farm-optimising decision-making along the way, in any context in which it is to be used. However, method evaluation, or prediction/decision validation, is also challenging. A range of measurements should be used to quantify the accuracy of predictions, which could include predictions of probability or severity as well as binary classifications for multiple health and welfare problems. For most data sets, accuracy needs to be measured appropriately to get around imperfect validation data, in particular to allow the monitoring method to detect the problem before it is recorded in the validation data. This is not straightforward, since the appropriate window size to use for this will likely depend on the type of problem, its severity, the recording method, and the monitoring data used.

Cross-validation is needed and should be applied to all quantitative methods in the same way, but how this is applied in detail is very important. Predictive accuracy can depend on the selected training and test set, particularly when either of them is small. Therefore, both full retraining and testing should be applied repeatedly with multiple random selections of training and test sets, and means and variation in performance should be reported. Variation needs to be low to provide evidence that methods are consistently reliable. To show methods generalise, cross-validation should be carried out with training and test sets selected made between individuals, or groups (e.g., batches or farms) where data is on multiple groups and spatial locations. Ultimately, methods need to be validated across many farms to show that they are practical for use on farms and to show any benefit from using them. Conventional experimental designs could be applied in multiple farm studies where the real impact of using sensors plus associated quantitative methods to generate alarms along with strict protocols for decision-making, could be evaluated based on their effect on usual farm animal performance, health, and welfare measurements as well as profit.

In conclusion, there is the potential to massively increase the use of empirical data for decision-making in real time, but the development and validation of quantitative approaches are still needed in order to move from the developmental stage to this intended practical application. Whilst there is wide-ranging literature in this area, in livestock farming there is a paucity that provides fair, robust statistical comparisons of alternative quantitative methods and evidence that the resulting decision-making performs adequately in practice. That is, it must be practically feasible to repeatedly apply the method dynamically in real time on farms and provide information of direct relevance to decision-making.

## Figures and Tables

**Table 1:** Examples of commonly used sensors for real-time monitoring of livestock on farms, showing types of sensors, what is being measured on what species, and the purposes of the monitoring.

Name	Species Being Monitored ^1^	What Is Being Measured	Sensor Technology	Purpose of Monitoring	Comments
1 Individual intakes	Dairy Cows, Beef Cattle, Calves, *Sheep*	Individual feeding and drinking behaviour, amounts if technology allows	Sensors at feeders/drinkers that record individual RFID tags. More advanced systems that also record feed or drink taken at each bout.	Managing Nutrition and Production; Detecting Health and Welfare Problems of individuals	
2 Group intakes	Dairy Cows, Beef Cattle, Calves, Pigs, Poultry, Goats, Sheep	Group feeding and drinking including amounts	Automatic livestock feeders and drinkers	Managing group Nutrition and Production; Detecting Health and Welfare Problems in groups	
3 Individual weights—identified individuals	Dairy Cows, Beef Cattle, *Pigs*, *Sheep*	Individual Live weights	Walk-over weighers that record individual RFID tags.	Managing Nutrition and Production; Detecting Health and Welfare Problems of individuals	Walk-over weighers placed to maximise the number of readings (e.g., on way in/out of milking parlour)
4 Individual weights—unidentified individuals	Pigs, Sheep	Live weights measured per individual but individuals not identified	Walk-over weighers (e.g., at races, or in pens)	Managing Nutrition and Production; Detecting Health and Welfare Problems in groups/of individuals	Can be used to sort into different feeding areas using marking and/or gate system. For pigs in pens, they can be placed between loafing and feeding areas or separated off if unwell.
5 Estimated weights—groups/ unidentified individuals	Poultry—Broilers, Turkeys	Live weight plus number on plate hence average liv weights; individuals not identified	Weighing Plates/Platforms for individuals/groups	Managing group Nutrition and Production; Detecting Health and Welfare Problems in groups	This is a sampling of weights in the flock. Could give 1000 s of weight measurements per day. Some platforms only measure one bird at a time whilst some measure multiple birds.
6 Milk parlour data	Dairy Cows	Milk yield, milking duration, peak flow; milk quality, Somatic Cell Count (SCC); position in parlour/milker	Automatic milking systems plus manual sampling	Managing Nutrition and Production; Detecting Health and Welfare Problems of individuals	Milk quality and SCC measures may not be available in real time but could be sampled regularly (e.g., once per day or week).
7 Milk bulk lab data	Dairy Cows	Somatic Cell Count (SCC), Milk quality	Milk bulk sampling—manual sampling	Managing group Nutrition and Production; Detecting Health and Welfare Problems in groups	Milk quality and SCC measures may not be available in real time but could be sampled regularly (e.g., once per day or week).
8 Milk bulk other data	Dairy Cows	Temperature, Volume, Stirring	Milk bulk sampling—various sensors	Managing group Milk Production and Processing	Real-time monitoring of physical attributes of milk in bulk tanks is available
9 Movement—acceleration	Dairy Cows, Beef Cattle, *Pigs*, *Sheep*, *Goat*	Behaviour (e.g., activity or time budgets in different classes: lying/standing, grazing/not, rumination, … or raw acceleration in x, y, and z directions)	Accelerometer	Detecting Heat, Calving/Lambing/Farrowing, Health and Welfare Problems	Not usually used on pigs on real farms. For sheep cheaper options needed. For grazing animals, they are often removed at intervals for data download and recharging. Can give raw accelerometer data but sometimes measures are derived only (e.g., behaviour).
10 Movement—gait	Dairy Cows, Beef Cattle, *Pigs*, *Sheep*, *Goat*	Behaviour (step count), other gait measurements	Pedometer	Detecting Heat, Calving/Lambing/Farrowing, Health and Welfare Problems	Less advanced than accelerometer; some just measure step count but others take measurements that can be used to detect lameness
11 Location	Dairy Cows, Beef Cattle, *Pigs*, *Sheep*, *Goat*	Location and behaviour	GNSS (Global navigation satellite system), GPS (global positioning system)	Managing Grazing and Production; Detecting Health and Welfare Problems of individuals	
12 Relative location	Dairy Cows, Beef Cattle, *Pigs*, *Sheep*, *Goat*	Location relative to static receivers and behaviour	Proximity loggers plus static receivers	Managing Grazing and Production; Detecting Health and Welfare Problems of individuals	Locations can be estimated as well as mother-offspring distances
13 Images—unidentified individuals	*Pigs*	Body condition score, liveweight	2D Imaging from above	Managing Nutrition and Production; Detecting Health and Welfare Problems of individuals	Can be placed between loafing and feeding areas and used to sort into different feeding areas using gate system
14 Images—identified individuals	*Cows*, *Pigs*	Body condition score, live weight, behaviour	2D/3D Imaging from above	Managing Nutrition and Production; Detecting Health and Welfare Problems of individuals	Identifying individuals is difficult, so it is used in combination with reading RFID tags at intervals and then tracking.
15 Images—unidentified birds	*Poultry*—*Broilers*, *Turkeys*	Location and behaviour; dead birds; weight estimation;	2D Imaging from above	Managing Nutrition and Production; Detecting Health and Welfare Problems of groups	Imaging systems for poultry tend to occur at a larger scale (per individual) than those for cows and pigs.
16 Temperature—unidentified individuals	*Dairy Cows*, *Beef Cattle*, *Calves*, *Pigs*, *Poultry*, *Goats*, *Sheep*	Body temperature	Thermal Imaging	Detecting Health and Welfare Problems of individuals	Can be used for detecting heat stress, and potentially fever, pain, …
17 Temperature—identified individuals	*Dairy Cows*	Body temperature	Thermometer	Detecting Health and Welfare Problems of individuals	
18 Sound—vocalisations	*Dairy Cows*, *Beef Cattle*, *Calves*, *Pigs*, *Poultry*, *Goats*, *Sheep*	Specific Species-Dependent Vocalisations	Acoustic Sensors	Detecting Health and Welfare Problems of Groups	These sensors are mounted in, e.g., house, but could be used outside in confined areas
19 Sound—feeding	*Cows*, *Sheep*	Feed intake, behaviour (grazing, ruminating)	Acoustic Sensors	Managing Grazing and Production	These sensors are mounted on animals
20 Aerial images—extensive	*Available Grazing for Cows*, *Sheep*	Quality of grazing	Remote Sensing (Satellite imaging)	Managing Grazing	
21 Aerial images—targeted	*Cows*, *Sheep and Available Grazing*	Quality of grazing; location of groups	Camera on Drone/UAV (Unmanned Aerial Vehicle)	Managing Grazing; Detecting Health and Welfare Problems	
22 Local environmental conditions	Livestock	Temperature, humidity, emissions (e.g., Ammonia, Methane, CO_2_)	Environmental sensors	Managing Health and Welfare Problems of groups; Managing emissions	Usually for housed livestock
23 Weather outside	Livestock	Temperature, humidity, Rainfall, Windspeed, …	Weather station	Managing Health and Welfare Problems of groups	Could affect housed livestock as well as livestock kept outside

^1^*Species shown in italics indicates when this monitoring tends to be for research studies currently rather than on real farms*.

**Table 2:** Examples (numbered as in Table 1) of commonly used sensors for real-time monitoring of livestock on farms showing various characteristics that may be associated with the measurements and resulting monitoring data streams. ● ● indicates that the monitoring data have the characteristic listed in the columns. The nature of the resulting sensor measurements is not listed here, as raw data may be unprocessed or may be processed in differing ways, leading to multiple types of measurements for each type of sensor that could be used for monitoring.

Name	Measurement On				Timing of Sensor Measurements			Animals Are		Sensor Is		Sensor Is	
	Individuals ID Known	Individuals ID Not Known	Some Individuals ID Not known	Groups or Impact on Groups	Continuous or Near-Continuous	Intermittent	Regular	Housed (Usually)	Outside/Grazing	On animal	Not on Animal	At Fixed Location ^1^	Mobile
1 Individual intakes	●					●		●	●		●	●	
2 Group intakes				●	●	●		●	●		●	●	
3 Individual weights—identified individuals	●					●	●	●	●		●	●	
4 Individual weights—unidentified individuals		●				●		●			●	●	
5 Estimated weights—group/unidentified individuals			●			●		●			●	●	
6 Milk parlour data	●					●	●	●	●		●	●	
7 Milk bulk lab data				●		●	●	●	●		●	●	
8 Milk bulk other data				●	●			●	●		●	●	
9 Movement—acceleration	●				●	●		●	●	●			●
10 Movement—gait	●				●	●		●	●	●			●
11 Location	●				●	●			●	●			●
12 Relative location	●				●	●			●	●		●	●
13 Images—unidentified individuals		●			●	●		●			●	●	
14 Images—identified individuals	●				●	●		●			●	●	
15 Images—unidentified birds			●	●	●	●		●	●		●	●	
16 Temperature—unidentified individuals		●			●	●		●	●		●	●	●
17 Temperature—identified individuals	●				●	●		●	●	●			●
18 Sound—vocalisations				●	●			●	●		●	●	
19 Sound—feeding	●				●	●			●	●		●	●
20 Aerial images—extensive				●		●			●		●		
21 Aerial images—targeted			●	●	●	●			●		●		●
22 Local environmental conditions				●	●			●			●	●	
23 Weather outside				●	●				●		●	●	

^1^ e.g., in milking parlour, at feeders, in field.

## Data Availability

No new data were created or analysed in this study. Data sharing is not applicable to this article. (Artificial data shown for illustrative purposes was simulated).

## Appendix A. Mathematical Details for Simulation Program

This appendix describes the statistical assumptions made in the simulation to generate sensor data.

Time series sensor data of length *T* timesteps from *S* types of sensors can be generated for I individuals, each in one of *G* management groups, with *H* types of illnesses (or health issues such as injuries or heat events). The time series sensor data is driven by the individual, management changes occurring for some or all individuals in a group, and health or welfare problems occurring over time for each individual. In order to cope with lags in associations between sensors and factors driving sensor data, all data are generated for time steps t=−τ,1−τ,2−τ,…,T+τ and then subsetted to t=1,2,…,T. However, for simplicity in the below we just refer to t=1,2,…,T.

### Appendix A.1. Generating Health Data

For illnesses, h=1,2,…,H, the probability any individual i=1,2,…,I can get illness *h* is $p_{h}$, so assigning a binary indicator, ${binh}_{hi}$, to be 1 for illness *h* and 0 for no illness *h*, for individual *i*,

(A1) $${binh}_{hi}\sim bernoulli(p_{h})$$

Given that an individual may have this illness, the probability of an illness episode centred around each time is $q_{h}$, so assigning a binary indicator, ${binh}_{hie}$, to be 1 for illness and 0 for no illness for individual *i* centred around time *e*

(A2)
$${binh}_{hie}\sim \left\{\begin{array}{c} bernoulli\left(q_{h}\right)\;if\;{binh}_{hi}=1\\ 0\;otherwise \end{array}\right.$$

Then for each illness episode, ${binh}_{hie}=1$, the true severity of the illness is generated as follows. A skew normal distribution is generated around the time of the episode, *e*, with parameters for variation and skew selected from two uniform distributions with ranges fixed dependent on the illness type, *h*. The resultant PDF is multiplied by a maximum severity and added to a baseline shift, both also generated from uniform distributions with ranges fixed dependent on the illness type, *h*.

(A3)
$$e{sev}_{hie}(t)=\delta_{hie}\left(t\right)\left({base}_{hie}+{{dosev}_{h}maxsev}_{hie}{pdf}_{hie}(t)\;\right)\mathrm{where}\;{pdf}_{hie}\left(t\right)=pdfskewN(0,\sigma_{hie},\gamma_{hie}),\;{dosev}_{h}=0\;or\;1,\;\delta_{hie}(t)=\left\{\begin{array}{c} 1\;if{pdf}_{hie}\left(t\right)>\delta_{h}\\ 0\;otherwise \end{array}\right.,\;\;\sigma_{hie}\sim uniform\left({\sigma low}_{h},{\sigma up}_{h}\right),\gamma_{hie}\sim uniform\left({\gamma low}_{h},{\gamma up}_{h}\right),\;{base}_{hie}\sim uniform\left({blow}_{h},{bup}_{h}\right),{maxsev}_{hie}\sim uniform\left({mlow}_{h},{mup}_{h}\right)$$

By choosing parameters appropriately, true severity could, for example, increase abruptly and then decrease more gradually, or increase gradually and then decrease abruptly, or could just be a step change up for a time, and then back down again later. The latter is achieved by setting, ${dosev}_{h}$ to 0 to indicate that there is only a base shift with length controlled by the normal skew distribution and a threshold, $\delta_{h}$.

A time series for the true severity for each illness and each individual is then constructed by summing over all the episodes for that individual

(A4)
$${sev}_{hi}\left(t\right)=\sum_{e}e{sev}_{hie}(t)$$

A binary indicator was also defined for whether an individual truly had illness *h* at time *t*, if the true severity exceeded a threshold, ${bs}_{h}$, differing between illness types.

(A5)
$${binsev}_{hi}\left(t\right)=\left\{\begin{array}{c} 1\;if{sev}_{hi}\left(t\right)>{bs}_{h}\\ 0\;otherwise \end{array}\right.$$

The observed severity was generated as a noisy linear function of the true severity, but also allowed for a lag dependent on the illness, ${lag}_{h}$,

(A6)
$${osev}_{hi}\left(t+{lag}_{h}\right)=\left\{\begin{array}{c} \delta_{hi}^{o}\left(t\right)\left(\alpha_{h}+\beta_{h}{sev}_{hi}\left(t\right)+\varepsilon_{hi}\right)\;if\;\delta_{hi}^{o}\left(t\right)>0\\ 0\;otherwise \end{array}\right.\mathrm{where}\;\delta_{hi}^{o}(t)=\left\{\begin{array}{c} 1\;if\;\left(\alpha_{h}+\beta_{h}{sev}_{hi}\left(t\right)+\varepsilon_{hi}>0\right)\;and\;\left(\delta_{h}^{o}=0\;or\;\left(\delta_{h}^{o}\left({sev}_{hi}\left(t+{lag}_{h}\right)\right)>0\right)\right)\\ 0\;otherwise \end{array}\right.\;\mathrm{and}\;\varepsilon_{hi}\sim N(0,\sigma_{h}^{2})\;\mathrm{and}\;\delta_{h}^{o}=0\;or\;1$$

Thus, if the lag, ${lag}_{h}$ is positive, there is a delay in the time that the animal is observed to be ill, and if the variation, $\sigma_{h}^{2}$ in a relationship, the observation process for the illness is not very accurate. Use of $\delta_{hi}^{o}(t)$ constrains the observed severity to be positive and allows an option that the observed severity can only be positive at times when the true severity is positive. The observation process for illnesses could be further altered by constraining the severity to only be observed (non-missing) at intervals ${int}_{h}$ (for example every 7 time steps) for some illnesses in some groups.

A binary variable was also defined for whether an individual was observed to have an illness at time *t*, if the observed severity exceeded a threshold, ${bos}_{h}$, based on the illness type *h*.

(A7)
$${binosev}_{hi}\left(t\right)=\left\{\begin{array}{c} 1\;if\;o{sev}_{hi}\left(t\right)>{bos}_{h}\\ 0\;otherwise \end{array}\right.$$

### Appendix A.2. Generating Management Data

For management groups, g=1,2,…,G, the probability of any exposure to management changes in group *g* is $p_{g}$, so, assigning ${bing}_{g}$ to be 1 for changes and 0 for no changes for group *g*,

(A8) $${bing}_{g}\sim bernoulli(p_{g})$$

And given that a group has any management changes, the probability of a change centred around each time is $q_{g}$, so assigning ${bing}_{gc}$ to be 1 for change and 0 for no change for group *g* centred around time *c*

(A9)
$${bing}_{gc}\sim \left\{\begin{array}{c} bernoulli\left(q_{g}\right)\;if\;{bing}_{g}=1\\ 0\;otherwise \end{array}\right.$$

Then for each change, ${bing}_{gc}=1$, a measurement of the management change (a management index) is generated analogously to the true disease severity as follows. A skew normal distribution is generated around the time of the change, *c*, with parameters for variation and skew selected from two uniform distributions with ranges fixed dependent on the group, *g*. The resultant PDF is multiplied by a maximum management index, and added to a baseline shift, both also generated from uniform distributions with ranges fixed dependent on the group, *g*. There is an option for a management change for each group to be positive or negative.

(A10)
$${cgman}_{gc}\left(t\right)={sgn}_{g}\delta_{gc}\left(t\right)\left({base}_{gc}+{{doman}_{g}maxman}_{gc}{pdf}_{gc}(t)\right)\mathrm{where}\;{sgn}_{g}=1\;or\;-1\;{pdf}_{gc}\left(t\right)=pdfskewN(0,\sigma_{gc},\gamma_{gc}),\;{doman}_{g}=0\;or\;1\;\delta_{gc}(t)=\left\{\begin{array}{c} 1\;if\;{pdf}_{gc}\left(t\right)>\delta_{g}\\ 0 \end{array}\right.,\;\sigma_{gc}\sim uniform\left({\sigma low}_{g},{\sigma up}_{g}\right),\gamma_{gc}\sim uniform\left({\gamma low}_{g},{\gamma up}_{g}\right),\;\;{base}_{gc}\sim uniform\left({blow}_{g},{bup}_{g}\right),{maxman}_{gc}\sim uniform\left({mlow}_{g},{mup}_{g}\right)$$

For individuals in groups, g=1,2,…,G, the probability of any management changes per individual in group *g* is $r_{g}$, so, assigning ${bing}_{gi}$ to be 1 for changes and 0 for no changes for individual *i* in group *g*,

(A11)
$$c{man}_{gic}\left(t\right)={{bing}_{gi}cgmanindex}_{gc}\left(t\right)\;\;where\;{bing}_{gi}\sim bernoulli(r_{g})$$

A time series for the management index for each individual is then constructed by summing over all the changes for that individual

(A12)
$${man}_{gi}\left(t\right)=\sum_{c}c{manindex}_{gic}\left(t\right)$$

Thus, the management index measurement is a numerical measurement that changes with time and is the same for all individuals in each group, though some individuals in a group may not be subjected to the change. By choosing parameters appropriately, the management index could, for example, increase abruptly and then decrease more gradually, or increase gradually and then decrease abruptly, or could just be a step change up for a time, and then back down again later. Or they could go down and then up and so on.

### Appendix A.3. Generating Sensor Data

Sensor data for each individual is assumed to be a linear combination at each time step of a baseline, adjustments due to any true illness of individuals, and adjustments due to management changes. This is implemented by forming 3 (unobserved) time series per individual and sensor type, which are then added together to obtain observed sensor data,

(A13)
$${sens}_{si}\left(t\right)={sensbase}_{si}\left(t\right)+\sum_{h=1}^{H}{senssev}_{shi}\left(t\right)+{sensman}_{sgi}\left(t\right)$$

For each type of sensor, s=1,2,…,S, baseline sensor time series data for each individual i=1,2,…,I. each in one of groups, g=1,2,…,G is generated using a hierarchical mixed model which allows for group-dependent variation between individuals, group-dependent variation between times within individuals, and individual-dependent variation between times within individuals, each of which is generated from a uniform distribution with parameters dependent on the sensor type,

(A14)
$${sensbase}_{si}\left(t\right)=\alpha_{s}+\beta_{sgi}+\beta_{sgi}\left(t\right)+\varphi_{sgi}\left(t\right)\mathrm{where}\;\beta_{sgi}\sim N\left(0,\sigma_{sg}^{2}\right),\beta_{sgi}\left(t\right)\sim N\left(0,\theta_{sg}^{2}\right),\varphi_{sgi}\left(t\right)\sim N\left(0,\vartheta_{sgi}^{2}\right)\mathrm{and}\;\sigma_{sg}^{2}\sim uniform\left({\sigma low}_{s},{\sigma up}_{s}\right),\theta_{sg}^{2}\sim uniform\left({\theta low}_{s},{\theta up}_{s}\right),\;\vartheta_{sgi}^{2}\sim uniform\left({\vartheta low}_{s},{\vartheta up}_{s}\right)$$

So, the baseline sensor time series data can have different means per type of sensor and individual, and then can be more or less variable within times dependent on the management group and on the individual.

The time series for each individual due to illnesses is generated, for each illness, as a noisy linear function of the true severity, but also allowing for a lag dependent on the illness and the type of sensor, ${lag}_{sh}$,

(A15)
$${senssev}_{shi}\left(t+{lag}_{sh}\right)=\delta_{shi}\left(t\right)\left(\alpha_{sh}+\beta_{sh}{sev}_{hi}\left(t\right)+\varepsilon_{shi}\right)\;\mathrm{where}\;\delta_{shi}(t)=\left\{\begin{array}{c} 1\;if\;\left(\delta_{sh}=0\;or\;\left({sev}_{hi}\left(t+{lag}_{sh}\right)>0\right)\right)\\ 0\;otherwise \end{array}\right.\;\mathrm{and}\;\varepsilon_{shi}\sim N(0,\sigma_{sh}^{2})\mathrm{and}\;\delta_{sh}=0\;or\;1$$

Thus, if the lag, ${lag}_{sh}$, is positive, there is a delay in the time that the individual sensor data changes in response to the individual being ill and if the variation, $\sigma_{sh}^{2}$, in the relationship, the large sensor is not well related to the severity of the illness. Use of $\delta_{shi}(t)$ allows an option that the sensor data can only be altered by the illness at times when the true illness severity is positive.

The time series for each individual due to management changes is generated as a noisy linear function of the management change index, but also allowing for a lag dependent on the group and the type of sensor, ${lag}_{sg}$,

(A16)
$${sensman}_{sgi}\left(t+{lag}_{sg}\right)=\delta_{sgi}\left(t\right)\left(\alpha_{sg}+\beta_{sg}{man}_{gi}\left(t\right)+\varepsilon_{sgi}\right)\;\mathrm{where}\;\delta_{sgi}(t)=\left\{\begin{array}{c} 1\;if\left(\delta_{sg}=0\;or\;\left({man}_{gi}\left(t+{lag}_{sg}\right)>0\right)\right)\\ 0\;otherwise \end{array}\right.\;\mathrm{and}\;\varepsilon_{sgi}\sim N(0,\sigma_{sg}^{2})\mathrm{and}\;\delta_{sg}=0\;or\;1$$

Thus, if the lag, ${lag}_{sh}$ is positive, there is a delay in the time that the individual sensor data changes in response to the management change and if the variation, $\sigma_{sg}^{2}$, in the relationship, the large sensor is not well related to the management index. Use of $\delta_{sgi}(t)$ allows an option that the sensor data can only be altered by the management change at times when the management index is positive.

### Appendix A.4. Parameters Used in Simulation

A single set of parameters and one run of the program were used in the simulation of data in this paper. The overall parameters used were

H=4, I=10, g=3, S=2, T=100, τ=100

The numbers of individuals in groups 1, 2, and 3 are 2, 4, and 6, respectively. Thus, sensor data from 2 types of sensors are being generated over 100 time steps for 10 individuals in one of 3 management groups, and 4 types of illnesses may occur for individuals. The remaining parameters are shown in Table A1, Table A2, Table A3, Table A4, Table A5 and Table A6, below.

Parameters used to simulate true illness severity (Table A1) have been chosen so that illness types have a possibility of occurring on average in 1 in 5 to 1 in 2 individuals, and with episodes centred on 1 in 100 to 3 in 100 timesteps on average per individual. Illness type 2 severity is just a step change up, whilst the other three illness types have skewed distributions with no baseline shift. Illness type 3 is shorter in duration than the rest. The binary variable for true illness is set so that it is 1 so long as the true severity is positive for all 4 illness types.

**Table A1:** Parameters used to simulate true illness severity for 4 types of illnesses.

Parameter Name	Equation	Parameters ^1^
$p_{h}$	(A1)	(0.4, 0.3, 0.5, 0.2)
$q_{h}$	(A2)	(0.01, 0.02, 0.03, 0.01)
${\sigma low}_{h}$	(A3)	(2.0, 1.0, 0.5, 2.0)
${\sigma up}_{h}$	(A3)	(4.0, 2.0, 0.5, 4.0)
${\gamma low}_{h}$	(A3)	(−0.5, −0.1, 0.1, −0.9)
${\gamma up}_{h}$	(A3)	(0.5, 0.1, 0.2, −0.4)
${blow}_{h}$	(A3)	(0, 10, 0, 0)
${bup}_{h}$	(A3)	(0, 30, 0, 0)
${mlow}_{h}$	(A3)	(20, 10, 20, 30)
${mup}_{h}$	(A3)	(50, 40, 25, 70)
${dosev}_{h}$	(A3)	(1, 0, 1, 1)
$\delta_{h}$	(A3)	(0.001, 0.001, 0.001, 0.001)
${bs}_{h}$	(A5)	(0, 0, 0, 0)

^1^ Dependent on type of illness, h=1,2,3,4.

Parameters used to simulate observed illness severity from true illness severity (Table A2) are chosen so that observed severity is assumed to be positively related to true severity for all illnesses, but with varying degrees of accuracy. Observed severity can only be positive when the true severity is positive for all 4 types of illnesses. For illness type 1, the observed accuracy lags 5 time units behind the true accuracy. For group 3, illness types 1 and 2 are only observed every 7 time units. The binary variable for observed illness is set so that it is 1 as long as the true severity exceeds the thresholds given for each illness type.

**Table A2:** Parameters used to simulate observed illness severity from true illness severity for 4 types of illnesses.

Parameter Name	Equation	Parameters ^1^
$\alpha_{h}$	(A6)	(0, 0, 0, 0)
$\beta_{h}$	(A6)	(0.5, 0.7, 0.7, 0.9)
$\sigma_{h}^{2}$	(A6)	(10, 5, 1, 4)
${lag}_{h}$	(A6)	(5, 0, 0, 0)
$\delta_{h}^{o}$	(A6)	(1, 1, 1, 1)
${int}_{hg}$	(A6)	((1, 1, 7), (1, 1, 7), (1, 1, 1), (1, 1, 1)) ^2^
${bos}_{h}$	(A7)	(10, 0, 5, 10)

^1^ Dependent on type of illness, h=1,2,3,4; ^2^ Dependent on type of illness, h=1,2,3,4 and on group, g=1,2,3.

Parameters used to generate the management index (Table A3) are set so management changes occur in all 3 groups, with changes centred on average on 1 in 100 timesteps. The management index for group 1 is just a step change down, whilst the other 2 groups have skewed distributions, 1 which goes up with a baseline shift and the other which goes down with no baseline shift. The management change in group 2 lasts for much longer than for groups 1 and 3 and the management index in group 2 is also more symmetric than the index for group 3. All individuals in groups 2 and 3 are subjected to the management changes whilst in group 1 only half of the individuals, on average, are subjected to the change.

**Table A3:** Parameters used to simulate management changes for 3 management groups.

Parameter Name	Equation	Parameters ^1^
$p_{g}$	(A8)	(1, 1, 1)
$q_{g}$	(A9)	(0.01, 0.01, 0.01)
${\sigma low}_{g}$	(A10)	(1, 20, 2)
${\sigma up}_{g}$	(A10)	(2, 20, 8)
${\gamma low}_{g}$	(A10)	(0.8, −0.1, 0.8)
${\gamma up}_{g}$	(A10)	(0.9, 0.1, 0.9)
${blow}_{g}$	(A10)	(20, 20, 0)
${bup}_{g}$	(A10)	(30, 30, 0)
${mlow}_{g}$	(A10)	(30, 30, 50)
${mup}_{g}$	(A10)	(50, 50, 60)
${doman}_{g}$	(A10)	(0, 1, 1)
${sgn}_{g}$	(A10)	(−1, 1, −1)
$\delta_{g}$	(A10)	(0.001, 0.001, 0.001)
$r_{g}$	(A11)	(0.5, 1.0, 1.0)

^1^ Dependent on management group, g=1,2,3.

Parameters used to generate the baseline sensor data per individual (Table A4) are set so the overall values for the first type of sensor are much higher and can be more variable between groups, between times within groups, and between times within individuals than the second type of sensor.

**Table A4:** Parameters used to simulate baseline sensor time series data for 2 types of sensors for individuals in one of 3 management groups.

Parameter Name	Equation	Parameters ^1^
$\alpha_{s}$	(A14)	(200, 100)
${\sigma low}_{s}$	(A14)	(10, 20)
${\sigma up}_{s}$	(A14)	(200, 40)
${\theta low}_{s}$	(A14)	(10, 5)
${\theta up}_{s}$	(A14)	(200, 30)
${\vartheta low}_{s}$	(A14)	(10, 5)
${\vartheta up}_{s}$	(A14)	(200, 10)

^1^ Dependent on sensor, s=1,2.

Parameters used to adjust sensor data due to true illness severity (Table A5) are chosen so that the adjustment is negative for all illnesses except illness type 3, which is positive for the first sensor, whilst the second sensor is unaffected. Adjustments in the first sensor are better related to the illness’s severity than adjustments in the second sensor. For illness type 1 the effect on the second sensor lags 5 time units behind the true illness severity.

**Table A5:** Parameters used to simulate sensor time series adjustment due to true illness severity for 4 types of illnesses.

Parameter Name	Equation	Parameters ^1^
$\alpha_{sh}$	(A15)	((0, 0, 0, 0), (0, 0, 0, 0))
$\beta_{sh}$	(A15)	((−2, −2, 2, −2), (−1, −1, 0, −1))
$\sigma_{sh}^{2}$	(A15)	((4, 4, 4, 4), (16, 16, 16, 16))
${lag}_{sh}$	(A15)	((0, 0, 0, 0), (5, 0, 0, 0))
$\delta_{sh}$	(A15)	((1, 1, 1, 1), (0, 1, 1, 1))

^1^ Dependent on type of sensor s=1,2 and type of illness, h=1,2,3,4.

Parameters used to adjust sensor data due to management changes (Table A6) are chosen so that the adjustment is positive for all groups, though when the management index goes down, so will the sensor data. Adjustments in the first type of sensor are more closely related to the management changes than the second sensor. For group 3 the effect on the second sensor lags 10 time units behind the management changes.

**Table A6:** Parameters used to simulate sensor time series adjustment due to management group changes for 3 management groups.

Parameter Name	Equation	Parameters ^1^
$\alpha_{sg}$	(A16)	((0, 0, 0), (0, 0, 0))
$\beta_{sg}$	(A16)	((1, 1, 1), (0.25, 0.25, 0.25))
$\sigma_{sg}^{2}$	(A16)	((1, 1, 1), (1, 1, 1))
${lag}_{sg}$	(A16)	((0, 0, 0), (0, 0, 10))
$\delta_{sg}$	(A16)	((1, 1, 1), (1, 1, 0))

^1^ Dependent on type of sensor s=1,2 and management group, g=1,2,3.

### Appendix A.5. Code Used

The simulation was implemented in R [187] using RStudio [188]. Coding was bespoke but used some basic R packages:

*data.table* [189] was used to stack data into long format. CRAN: Package data.table (https://cran.r-project.org/web/packages/data.table/index.html, accessed on 7 September 2025)

*dplyr* [190] was used to find the times of first maxima in time series data. CRAN: Package dplyr (https://cran.r-project.org/web/packages/dplyr/index.html, accessed on 7 September 2025)

*ggplot* from *ggplot2* [191] was used to plot graphs. CRAN: Package ggplot2 (https://cran.r-project.org/web/packages/ggplot2/index.html, accessed on 7 September 2025)

*ggarrange* from *ggpubr* [192] was used to arrange multiple graphs in one frame. CRAN: Package ggpubr (https://cran.r-project.org/web/packages/ggpubr/index.html, accessed on 7 September 2025)

*sn* [193] was used to generate data from skewed normal distributions. CRAN: Package sn (https://cran.r-project.org/web/packages/sn/index.html, accessed on 7 September 2025)
